# Living within: endosymbiosis in parasites of medical importance—profits, expenses, and implications for host–parasite interactions

**DOI:** 10.3389/fcimb.2026.1956183

**Published:** 2026-10-12

**Authors:** Iman R. Abdel Shafi, Safeya O. El Bassiouni, Magda S. A. Abdeltawab

**Affiliations:** Medical Parasitology Department, Faculty of Medicine, Cairo University, Cairo, Egypt

**Keywords:** endosymbionts, host-parasite interaction, bacteria, viruses, parasitic infections, viral-like particles

## Abstract

Endosymbiosis is a biological relationship, where one organism lives within the body of another. The concept of endosymbiosis was first introduced relevant to the origin and development of organelles in eukaryotes. Later, microorganisms were reported to be associated with different eukaryotes including arthropods, parasitic helminths, and protozoa; subsequently, their role in parasite biology and parasite–human host interactions has been minded. Within this context, filaria–*Wolbachia*, *Leishmania*–*Leishmania* RNA virus (LRV), and *Trichomonas*–*Trichomonas vaginalis* virus (TVV) are extensively studied models that demonstrate how endosymbionts can directly drive immunomodulation in human host and worsen the clinical outcome, whereas *Acanthamoeba* stands out as a reservoir of an array of microorganisms including pathogenic bacteria, and a training hub for bacterial virulence evolution. Despite the progress achieved in understanding these models, the depth of characterization of endosymbionts across parasite taxa is markedly uneven. Disclosing the world of microorganisms living within parasites, especially with the advanced metagenomic analysis, constitutes an emerging field of research of immense scientific significance for parasitology, microbiology, and host–parasite interactions.

## 1 Introduction

Human parasitic infections have long represented an intractable problem in global public health. Although significant progress has been achieved in the treatment and control of medically important parasites, they continue to have considerable morbidity impacts and socioeconomic burden especially in developing countries (Ung et al., 2021). Meanwhile, knowledge and gaps on parasite biology, virulence factors, vulnerabilities, and pathogenicity remain to be elucidated.

Endosymbiosis is a biological relationship, where one organism lives within the body of another. The concept of endosymbiosis was first introduced relevant to the origin and development of organelles in eukaryotes, mainly mitochondria and plastids, which were contemplated to be acquired from bacterial endosymbionts (Taylor et al., 2012**;** Manoj et al., 2021). Then, evidence of endosymbiotic bacteria and fungi within arthropods was established, and later microorganisms were reported to be associated with different eukaryotes, including parasitic helminths and protozoa (Afzelius et al., 1989**;** Taylor et al., 2012). Virus-like particles (VLPs) were observed in parasites in the 1960s, and a molecular proof of viruses within protozoa was documented in the late 1980s, leading to the uncovering of double-stranded RNA (dsRNA) viruses in a number of parasitic protists (Molyneux, 1974**;** Benchimol et al., 2002**;** Barrow et al., 2020). Over the past years, an increasing array of microorganisms has been identified within parasites (**Figure 1**).

**Figure 1:**
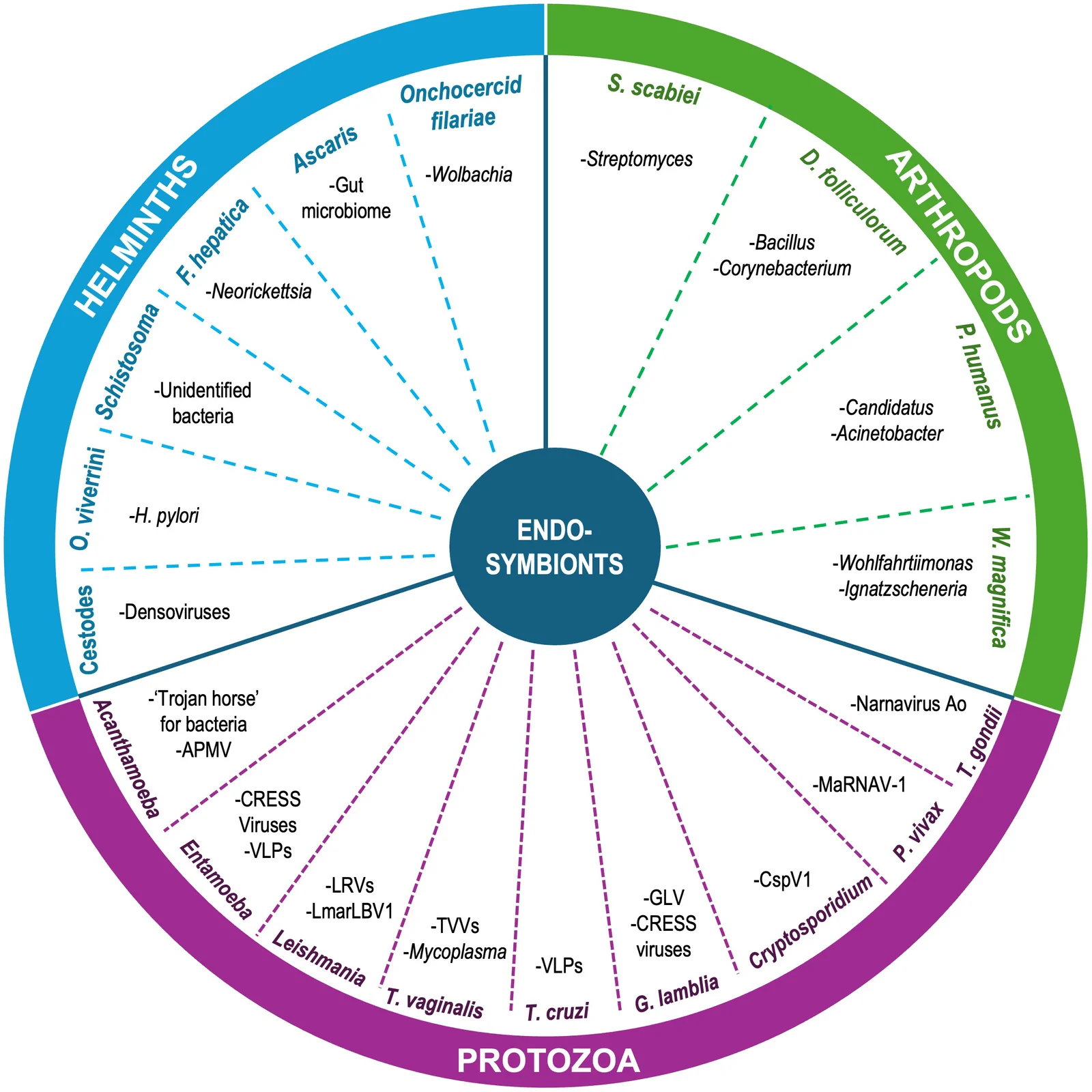
Parasite–endosymbiont overview. Microbial communities identified within different parasite taxa.

It is important to note that symbiosis is a complex phenomenon where the biological relationships span over a continuum ranging broadly from parasitism to commensalism and to mutualism. However, modern consensus has highlighted the overlap between these distinct terms and their highly dynamic context-dependent nature, as microbial behavior can shift according to ecological composition and environmental conditions within the host, as well as evolutionary time (Raina et al., 2018**;** Wiles and Guillemin, 2019). In fact, studies on the parasite–endosymbiont association were first of evolutionary concerns. More recently, researchers have paid attention to the role of this association in parasite biology and parasite–human host interactions. Research on parasitic helminth microbiomes remains scarce, and knowledge on the incidence and the impacts of the harbored microorganisms is still being developed (Jenkins et al., 2019**;** Gobert et al., 2022). Many pathogenic bacteria have been found to be infected with lysogenic bacteriophages that markedly affect their virulence, while information regarding protozoan endoviruses is lagging far behind (Janssen et al., 2015**;** Lafleur and Olivier, 2022).

Within the scope of this review, the term “symbiosis” is used to describe the association between the parasite and the microorganisms residing within it, whereby neither partner is harmed and one or both may benefit from the association (Martin and Schwab, 2013). This narrative review synthesizes current knowledge of parasite microbiome across medically important protozoa, helminths, and arthropods, with particular emphasis on its role within the host–parasite–endosymbiont triad. The objective is to provide a framework for understanding parasite–microbial interactions and their impact on host–parasite interactions, while highlighting their potential as therapeutic targets.

## 2 Methodology

To construct this narrative review, we systematically searched multiple databases: PubMed, Scopus, and Google Scholar. No date limit was applied on the literature search to retrieve all relevant studies including early foundational publications. We focused on endosymbiotic associations in key parasites infecting humans. Search terms included the following: “*Acanthamoeba*”, “*Entamoeba*”, “*Giardia*”, “*Leishmania*”, “*Trypanosoma*”, “*Trichomonas*”, “*Cryptosporidium*”, “*Plasmodium*”, “*Toxoplasma*”, “*Blastocystis*”, “filarial nematodes”, “*Wuchereria*”, “*Onchocerca*”, “intestinal nematodes”, “*Fasciola*”, “*Schistosoma*”, “*Opisthorchis*”, “Flat worms”, “Cestodes”, “*Echinococcus*”, “*Taenia*”, “*Hymenolepis*”, “*Sarcoptes*”, “*Demodex*”, “*Pediculus*”, and “*Wohlfahrtia*”, as well as “Endosymbiont”, “Endosymbiosis”, “Endosymbiotic bacteria”, “Bacterial endosymbiont”, “Endosymbiotic virus”, “Viral endosymbiont”, and “Viral-like particles”. We incorporated the Boolean queries “AND” and “OR” as appropriate to the above terms to generate the search strategy. The scope of this review encompassed primary and secondary research of heterogenous evidence types, focusing on endosymbionts or microbiota harbored within medically important parasites and exploring their potential impact on the parasite biology and the host–parasite interactions. Studies investigating endosymbiosis in parasite species that do not infect humans; studies involving hyperparasites, endosymbiont organelles, and evolutionary symbiosis; and non-peer-reviewed preprints were excluded.

Literature selection followed a standardized two-stage screening process: title/abstract screening followed by full-text evaluation. Extracted data points included taxonomic classification of the parasite species and the associated endosymbiont, technique employed, endosymbiont impact on the parasite biology, and host–parasite–endosymbiont crosstalk. Findings were organized according to parasite taxonomy integrating data from review articles, human observational studies, and experimental studies. Given the narrative nature of this review, we acknowledge that the scope of the search was non-exhaustive. Nevertheless, studies were curated through the authors’ judgment to provide a representative and significant overview of the relevant literature.

## 3 Endosymbionts of parasitic protozoa

### 3.1 Amoebozoan parasites

*Acanthamoeba* (Lobosa) is a free-living amoeba that can act as an opportunistic human pathogen. It has been identified from different environmental sources and can cause fatal granulomatous encephalitis and blinding keratitis [“*Acanthamoeba* keratitis” (AK)] (Siddiqui and Khan, 2012**;** Hajialilo et al., 2019). Among *Acanthamoeba* genotypes (T1–T23), T4 is the most prevalent in environmental samples and the most commonly involved in human infections (Hajialilo et al., 2019**;** Rayamajhee et al., 2022). The amoebae have been reported to host a variety of organisms comprising bacteria, viruses, protists, and yeasts (Siddiqui and Khan, 2012). *Acanthamoeba* has been described as the “Trojan horse” of microorganisms; trophozoites and cysts can maintain microorganisms and act as reservoir of human pathogens. Various bacterial groups were found to be carried by *Acanthamoeba* spp., including *Legionella pneumophila*, *Aeromonas* spp., *Escherichia coli*, *Pseudomonas aeruginosa*, and *Shigella* spp., *Vibrio cholerae* (Mungroo et al., 2021), as well as *Mycobacteria*, *Chlamydia* spp., *Rickettsia* spp., *Coxiella* spp., and *Bartonella* spp. (Price et al., 2024). The majority of *Acanthamoeba* clinical isolates from patients with AK were found to carry one or more bacterial endosymbionts that were recorded to affect corneal pathology and disease outcome (Rayamajhee et al., 2022). Mungroo et al. (2021) revealed that *Acanthamoeba*-associated microorganisms use amoeba as a shield to resist stressful conditions and evade the human host immune response, while multiplying safely and adapting and exchanging genes within, thus leading to potentiated pathogenicity. Furthermore, Price et al. (2024) proposed that phagocytic predatory amoebae serve as “training ground” where evolution and selection of microorganisms occur, thus potentiating their pathogenic capabilities. In the early 2000s, a new virus called *Acanthamoeba polyphaga* Mimivirus (APMV) was isolated. The virus has a large and complex double-stranded DNA (dsDNA) genome, exceeding the size of many bacteria, and a new family was created—the Mimiviridae (Colson et al., 2013). Later, numerous giant viruses (phylum Nucleocytoviricota) associated with *Acanthamoeba* spp. were identified (Queiroz et al., 2022). The detection of giant viruses of amoebae in humans and the study of their possible virulence are emerging research areas (Colson et al., 2017). APMV is an assumed pneumonia agent detected in sputum and bronchoalveolar lavage samples (Sakhaee et al., 2022); its role in nosocomial infections is still under investigation (Bousbia et al., 2013). Regarding amoeba–giant virus interaction, the virus can prevent amoebal encystation through inhibition of serine proteinase expression, a protein essential for the process. Meanwhile, *Acanthamoeba* cysts are reported to be resistant to APMV infection as long as the stimulus for cell encystment started before the viral infection (Boratto et al., 2015**;** Queiroz et al., 2022).

*Entamoeba histolytica* (Conosa) is the causative agent of amoebiasis, a parasitic infection of great public health concern in developing countries. Approximately 10% of infected individuals develop an invasive disease. Details regarding the pathobiological aspects of tissue invasion by the parasite in intestinal and extraintestinal diseases are still not clear (Guillén, 2023). Protozoal endoviruses were demonstrated for the first time in *E. histolytica*, following electron microscopy observation of VLPs that caused parasite lysis in axenic cell cultures (Diamond et al., 1972). A series of studies then described the morphogenesis of the amoeba viral particles, but the taxonomy of the virus had not been determined (Mattern et al., 1977**;** Hruska et al., 1974). No further investigation on the subject of *Entamoeba* virus genome sequences was accomplished as reported by Lafleur and Olivier (2022). Fifty years later, Kinsella et al. (2020) implicated *Entamoeba* and *Giardia* parasites as hosts of CRESS viruses. CRESS viruses are a group of small single-stranded DNA (ssDNA) viruses having a circular conserved replication-associated protein (REP) and a diverse host range (Zhao et al., 2019). Unclassified CRESS viruses are frequently detected in human stool samples, raising questions regarding host specificity and clinical significance (Phan et al., 2015). Using metagenomic sequencing, two CRESS virus families, Naryaviridae and Nenyaviridae, were reported to infect *Entamoeba*, while *Giardia* was infected by the Vilyaviridae family (Kinsella et al., 2020). The CRESS virus family Redondoviridae was reported to be linked to human periodontal disease and critical illness. Redondovirus and the oral commensal amoeba *Entamoeba gingivalis* coexist in metagenomic data from oral disease and healthy human subjects (Abbas et al., 2019). Keeler et al. (2023) investigated the cell niche of the Redondovirus in oro-respiratory specimens and recorded *E. gingivalis* as the potential host supporting its replication.

### 3.2 Excavata parasites

*Leishmania* spp. (Discoba) are intracellular trypanosomatid parasites, occurring in two subgenera *Leishmania* and *Viannia*, and are transmitted by phlebotomine sandflies (de Almeida et al., 2017). Depending on the *Leishmania* spp., three main clinical forms of leishmaniasis occur: visceral (VL), cutaneous (CL), and mucocutaneous (MCL). Worldwide, over 12 million individuals are infected with *Leishmania* spp. (Lockard et al., 2019). VLPs were first detected in *L. hertigi* (a lizard parasite), and later *Leishmania* dsRNA viruses (LRVs) were reported to inhabit the parasite as a natural host (Saberi et al., 2019). LRVs (family Totiviridae/genus *Leishmaniavirus*) are non-enveloped dsRNA viruses classified into two main types: LRV1 and LRV2. The LRV1 type comprises the New World (NW) strains (*L.V. guyanensis* and *L.V. braziliensis*) and is divided into 14 subtypes. The Old World (OW) strains (*L. major*, *L. tropica*, and *L. aethiopica*) belong to the LRV2 type (Zangger et al., 2014**;** Ginouvès et al., 2016**;** Cantanhede et al., 2018**;** Barrow et al., 2020). LRV is transmitted vertically in the parasite during multiplication, and as VLPs were detected in *Leishmania* exosomes, it may also be horizontally conveyed (Atayde et al., 2019). LRV was noted to promote parasite-infected mammalian cell survival through AKT1 prosurvival kinase phosphorylation (Eren et al., 2016). LRVs were found not to directly affect the parasite growth. The release of the virus from the degraded parasite impairs the innate immune response by inducing hyperinflammation via Toll-like receptor (TLR3) signaling activation. Thus, the virus can induce increased parasite burden, disease exacerbation, metastatic disease, and parasite persistence (Hartley et al., 2012**;** 2016**;** Parmentier et al., 2016**;** Rossi and Fasel, 2018). Furthermore, LRV was proposed to enhance expression of the parasite key virulence factors GP63 and MPI, allowing *Leishmania* to evade digestion in macrophages (Mirabedini et al., 2023**;** Sabeti et al., 2025). A new non-LRV *Leishmania* endosymbiont virus has been discovered that is hosted by *L. martiniquensis*, a species that can cause severe visceral disease in humans. The virus was found to have several features of the Bunyaviridae, which are enveloped, negative-sense ssRNA viruses, and this Leishbunyavirus was termed LmarLBV1 (Grybchuk et al., 2020). *Leptomonas* spp. (monoxenous trypanosomatids) are regarded nonpathogenic to man; meanwhile, *Leptomonas seymouri* was reported as a co-infectant in clinical specimens from VL and PKDL cases (Singh et al., 2013). Thakur et al. (2020) proclaimed that *Leptomonas* spp. can act as opportunistic human pathogens. A *L. seymouri* narna-like virus, the Lepsey NLV1 naked positive ssRNA virus, was demonstrated in Indian VL cultured isolates, raising the question of its possible role in disease pathogenicity (Sukla et al., 2022). In contrast to their relatives within the same clade, the human-infecting *Trypanosoma brucei* and *Trypanosoma cruzi*, responsible for Human African Trypanosomiasis and Chagas disease, respectively, are described as endosymbionts lacking trypanosomatids (Alves et al., 2013). However, in a study by Fernández-Presas et al. (2017), clusters of enveloped and non-enveloped VLPs were observed within *T. cruzi* epimastigotes using electron microscopy, though reported to be absent from the amastigotes and trypomastigotes. Their acquisition, characterization, and biological significance require further investigation.

*Trichomonas vaginalis* (Metamonada) is an obligate parabasalid flagellated parasite of the human genitourinary tract and the agent of a widespread sexually transmitted inflammatory disease (Zhang et al., 2023). *T. vaginalis* has a remarkable ability to establish symbiotic relationships with other microorganisms (Mercer and Johnson, 2018**;** de Aquino and Simoes-Barbosa, 2022). In 1985, a dsRNA virus of the Totiviridae family was found to live within *T. vaginalis*, the first virus discovered in a protozoan organism (Graves et al., 2019a). *Trichomonas vaginalis* virus (TVV) has four distinct species and is closely associated with the parasite Golgi complex (Barrow et al., 2020). Manny et al. (2022) reported a new TVV species using transcriptome mining (TVV5). Approximately 40% to 100% of *T. vaginalis* isolates shelter the virus, with TVV1 being the most commonly encountered (Fichorova et al., 2017). TVV endosymbiont has been demonstrated to upregulate the expression of *T. vaginalis* major immunogenic proteins, the cysteine proteinases that are linked to cytotoxicity, cytoadherence, and host immune evasion. Additionally, TVV has been associated with varying compartmental expression of P270, another *T. vaginalis* immunogenic protein, yet its role in pathogenicity remains unclear (Fichorova et al., 2013**;** He et al., 2017**;** Rada et al., 2022**;** Graves et al., 2025). TVV particles are liberated from the parasite within secretory extracellular vesicles (sECVs), which are reported to stimulate a higher proinflammatory response compared to non-infected *T. vaginalis* strains (Rada et al., 2022). TVV1 and TVV2 have been linked to trichomoniasis severity, whereas TVV2 and TVV3 have been associated with parasite virulence factor expression (Graves et al., 2019b). In addition to TVV, *Mycoplasma hominis* was identified in *T. vaginalis* clinical isolates by PCR in 1998. Most *T. vaginalis* isolates were found later to harbor *M. hominis*, and a novel species called *Candidatus Mycoplasma girerdii*. *Mycoplasma* is a member of the bacterial class Mollicutes, very small bacteria that lack a cell wall; *M. hominis* is a potential pathogen involved in bacterial vaginosis, and its strict symbiosis with *T. vaginalis* has been reported to be a unique association of two obligate human parasites (Dessì et al., 2019). *M. hominis* multiplies readily within the trichomonad, which provides a safe environment for the bacterium and plays a role in its transmission. In return, *Mycoplasma* can affect *T. vaginalis* growth rate, and its biochemical metabolic pathways promote a mutual benefit. *T. vaginalis*–*Mycoplasma* alliance can interfere with nitric oxide production by macrophages by competing for arginine, the key substrate during the immune reaction (Margarita et al., 2016**;** Fichorova et al., 2017). The bacteria were also shown to modulate *T. vaginalis* gene expression and to enhance its cytoadherence and hemolytic activity *in vitro* (de Aquino and Simoes-Barbosa, 2022). *T. vaginalis*–*Mycoplasma* endosymbiosis potentiates the trichomonad vaginal pathogenesis by upregulation of the human host proinflammatory response to the parasite, and predisposing to the outcomes associated with severe inflammation (Dessì et al., 2019). More recently, *T. vaginalis*–*Mycoplasma* endosymbiosis has been shown to modulate host immune signaling pathways; the presence of *M. hominis* drives type 1 IFN responses mediated by IFN-ϵ, which protect against cytoadherence and host cell death. This protective effect is disrupted by *T. vaginalis* sECVs that downregulate type 1 IFN gene expression, promoting host cell lysis and increasing parasite burden (Kochanowsky et al., 2025).

*Giardia lamblia* (Metamonada) is a diplomonad flagellate designated among the most prevalent intestinal protozoan pathogen in humans worldwide (Adam, 2021). Eight different genetic assemblages of the parasite species complex, A through H, are recognized. Assemblages A and B are infectious to humans, while assemblages C to H are related to infections in animals (Caccio et al., 2018). *G. lamblia* was reported to be a niche for an endosymbiotic dsRNA virus in the 1980s, the Giardiavirus (GLV) classified as Totiviridae (Janssen et al., 2015). Unlike other Totiviridae, GLV is more thermoresistant, has a different capsid protein translation site, and can be extracellularly transmitted (Garlapati and Wang, 2004). GLV seems to infect the trophozoite through receptor-mediated endocytosis and to leave it without cell lysis. The growth of the parasite was found to be arrested when GLV reaches a high titer, approximately 500,000 viruses per trophozoite (Lagunas-Rangel et al., 2021). A *Giardia* chaperone protein (GdDnaJ) has been suggested as a potential regulator of *G. lamblia* proliferation and GLV replication (Zhang et al., 2023). Several studies have revealed a virus infection rate of approximately 30% in *Giardia* isolates of different assemblages (Miska et al., 2009; Barrow et al., 2020). Marucci et al. (2021) indicated the occurrence of at least two GLV subtypes, which display distinct phenotypes and transmissibility in experimental infections of a GLV naïve *Giardia* isolate. The biological significance of parasite–GLV endosymbiosis and its impact on refractory giardiasis and treatment failure remain to be elucidated.

### 3.3 Alveolata parasites

*Cryptosporidium* (Apicomplexa) is a coccidian parasite causing severe diarrhea in children and immunocompromised patients (Gerace et al., 2019). The parasite has over 35 species with various host specificities, with *C. hominis* and *C. parvum* primarily infecting humans (Mkandawire and Sateriale, 2022). On the basis of gp60 gene, the most polymorphic marker in the *Cryptosporidium* genome, *C. hominis* and *C. parvum* sequences have been subtyped (Ia–Ig for *C. hominis* and IIa–IIo for *C. parvum*) (Rojas-Lopez et al., 2020; Lebbad et al., 2021). *Cryptosporidium* initiates frequent outbreaks around the world, and meanwhile, there is no effective treatment for the infection, mainly due to research impediments including efficient *in vivo* and *in vitro* models (Marzook and Sateriale, 2020). *Cryspovirus* (CspV1), a new genus in the family Partitiviridae non-enveloped dsRNA viruses, was first found to infect *C. parvum* in 1997 (Adjou et al., 2023). More than one species of the virus is speculated, as sequence-based divergence between CspV1 from *C. parvum* and viruses from *C. hominis, C. felis*, and *C. meleagridis* has been revealed (Jenkins et al., 2015). CspV1 can positively affect parasite fecundity with higher multiplication rates interpreted in culture studies (Jenkins et al., 2008; Lafleur and Olivier, 2022). The virus is transmitted intracellularly within the oocyst stage during cell division. CspV1 sequence detection was deduced to have a great potential as a tracing marker for *Cryptosporidium* infection sources (Adjou et al., 2023). *Cryptosporidium* can exploit the virus–endosymbiont association for evasion of the host immune response, through activation of type 1 IFN response and attenuation of IFN-γ-mediated epithelial antiparasitic defense (Deng et al., 2023). Graham et al. (2023) reported that *C. parvum* abducts a host long noncoding RNA (LncRNA) U90926 to evade the intestinal epithelial immune reaction, and CspV1 was found to upregulate U90926 during the infection.

*Toxoplasma gondii* (Apicomplexa) is a ubiquitous coccidian parasite in the family Sarcocystidae. *T. gondii* displays great genetic diversity, and strains have been classified into archetypal (I, II, and III genotypes) and non-archetypal strains (Bastilho et al., 2025). *T. gondii* infection persists as a hazardous opportunist to immunocompromised patients and pregnant women; moreover, it is a leading infectious cause of uveitis (Goh et al., 2023). From a computational viral screen of human neuronal datasets, Gupta et al. (2024) reported a novel narnavirus (*Apocryptovirus odysseus* Ao) associated with two hypervirulent strains of *T. gondii*—RUB and COUGAR. The narnavirus Ao is a member of the highly divergent Narnaviridae group of naked positive-sense RNA viruses. The authors presented an *in silico* evidence that Ao is a plausible proinflammatory inducer in human and mouse cells infected with *T. gondii* and suggested that Ao could function as a hypervirulence factor.

*Plasmodium vivax* (Apicomplexa) is a haemosporidian parasite, which is the main *Plasmodium* species causing malaria outside of sub-Saharan Africa (Mazhari et al., 2023). *P. vivax* has a strict host tropism for reticulocytes (Gruszczyk et al., 2018). The parasite can form a latent liver stage, the hypnozoite, which later activates and triggers a disease relapse (Schäfer et al., 2021). Through a metatranscriptomic investigation of blood samples from patients with malaria infected with *P. vivax, P. falciparum*, and *P. knowlesi*, a Narnavirus-like sequence was discovered in *P. vivax*-infected patients and not in other patients with malaria. A new single-stranded RNA virus, termed Matryoshka RNA virus 1 (MaRNAV-1), was identified. As the virus was found to be prevalent in geographically diverse *P. vivax* isolates obtained from humans and mosquitoes, a strong association of MaRNAV-1 and *P. vivax* was speculated (Charon et al., 2019). Currently, ongoing research aims to answer unresolved questions regarding the emergence and evolution of this specific virus–parasite association, which is absent in other *Plasmodium* species infecting humans including the hypnozoite-forming species *P. ovale*. In addition, the role of MaRNAV-1 in *P. vivax* transmission potential, pathobiology, and disease severity is still being investigated (Petrone et al., 2026).

### 3.4 Stramenopiles parasite

*Blastocystis* spp. is considered one of the most common intestinal protozoa detected in humans, mammals, and birds, encompassing at least 40 subtypes (ST) based on SSU-rRNA analysis (Zhang et al., 2025). *Blastocystis* ST1 to ST9 are found in human colon and regarded as dynamic yet enigmatic elements in the human gut microbial community. Despite the wide spread of *Blastocystis* infection and the advances in subtype identification, literature on *Blastocystis* spp. clinical significance is often contradictory. There were subtype-specific variations regarding the effect of *Blastocystis* on gut functions, where both beneficial and harmful modulations of the intestinal microenvironment have been reported. Regarded as having a context-dependent dual effect, *Blastocystis* spp. have been reported to disrupt the intestinal epithelial barrier and induce gut dysbiosis, thereby augmenting GIT inflammation. Conversely, they have also been found to exert anti-inflammatory effects by maintaining a balanced microbial composition and preserving intestinal barrier integrity (Deng and Tan, 2025). Shaw et al. (2026) proposed the possible presence of bacterial and VLP endosymbionts in *Blastocystis* spp. after reviewing the limited reports published over the past 50 years, which paves the way for further testing and confirmation.

## 4 Endosymbionts of parasitic helminths

### 4.1 Filarial nematodes

Nematodes of the family Onchocercidae (Spirurida/Filarioidea) are the causative agents of lymphatic filariasis (LF) and onchocerciasis, two diseases representing major health issues. They are mainly found in tropical and subtropical regions of the developing world and are transmitted by blood-feeding insects (Ramaiah and Ottesen, 2014). Late in the 1960s and early 1970s, rickettsia-like intracellular particles were first detected in filarial nematodes, and later identified as *Wolbachia* bacteria (Ferri et al., 2011). Onchocercid worms have a striking dependence on the endosymbiotic bacterium *Wolbachia* (Taylor et al., 2005).

*Wolbachia* is a Gram-negative alphaproteobacterium belonging to the order Rickettsiales. The bacteria infect two groups of organisms: arthropods, where they are widespread, infecting approximately 40% of terrestrial arthropods, and a single family of nematodes, the Onchocercidae (Zug and Hammerstein, 2012; Bouchery et al., 2013). *Wolbachia* has been classified into different molecularly identified supergroups, all attributed to one species, *W. pipientis*; supergroups C and D are limited to filariae while supergroup F encloses both arthropod and filarial hosts (Casiraghi et al., 2005). The bacteria were found in the filarial species causing LF: *Wuchereria bancrofti*, *Brugia malayi*, and *B. timori*; onchocerciasis: *Onchocerca volvulus*; mansonellosis: *Mansonella perstans* and *M. ozzardi*; and zoonotic dirofilariasis: *Dirofilaria immitis* and *D. repens*, but not in *Loa loa* (Hübner et al., 2022). *Wolbachia* has a wide range of symbiotic relations with its hosts. Its symbiosis spectrum extends from mutualistic and essential associations in filariae, where *Wolbachia* contributes to biosynthesis of molecules necessary for host fertility and viability such as heme, riboflavin, and nucleotide synthesis, to facultative and parasitic associations in the majority of infected arthropods (Manoj et al., 2021). *Wolbachia* is localized mainly in the female reproductive system and the hypodermal lateral cord in the infected onchocercid worms, it is found in all larval stages and is transmitted through the worm germ line to the next progeny (Ferri et al., 2011). The bacteria vary in density within the tissues of different onchocercids, and between life cycle stages and adult male and female of the same species (Fenn and Blaxter, 2004**;** Manoj et al., 2021). *Wolbachia* endosymbionts are reported to be involved in filarial worm embryogenesis, molting, and growth. *Wolbachia* was reported to provoke innate and adaptive TH1 inflammation responses, which can either be involved in filarial disease pathogenesis or promote infectivity through recruitment of mast cells that alter the blood flow and help infective larvae entry (Turner et al., 2006**;** Setegn et al., 2024). The concept of using anti-*Wolbachia* drugs as a treatment option of filarial diseases has been adopted due to its several advantages. The antibiotic treatment eliminates the bacteria and interrupts embryogenesis and microfilariae (MF) production, leading to permanent sterilization of female worms; meanwhile, the drugs achieve significant macrofilaricidal effect. In addition, as the anti-*Wolbachia* drugs do not induce rapid clearing of MF from the circulation or skin, they do not bear the risk of serious adverse incidents (Hübner et al., 2022). Breaking the nematode–bacterium association, by targeting either the bacteria or their molecular interactions, was claimed to reduce the risk of antibiotic resistance (Wangwiwatsin et al., 2023) and might achieve filarial infection elimination through downregulation of MF chitinase enzyme essential for its exsheathment and subsequent development in the vector (Quek et al., 2022).

### 4.2 Intestinal nematodes

Intestinal nematodes that commonly infect humans include the soil-transmitted helminths, the hookworms*, Ascaris lumbricoides, Trichuris trichiura*, and *Strongyloides stercoralis*, in addition to *Enterobius vermicularis*. The soil-transmitted helminths are part of the neglected tropical diseases that are prevalent in the disadvantaged communities causing chronic debilitation and impaired development in children (Stepek et al., 2006**;** Hassan et al., 2022). In contrast to the well-established *Wolbachia* spp. endosymbiont model in filarial nematodes, microbiome within intestinal nematodes has not been extensively investigated. Recent research explored *A. lumbricoides* gut microbiota and compared it to human gut microbiota using a metagenomic approach. *A. lumbricoides* gut microbiome was found to be distinct from human host gut microbiome; furthermore, *A. lumbricoides* microbiome population was demonstrated to differ according to worm burden within the host (Klomkliew et al., 2022; Muslim et al., 2024).

### 4.3 Platyhelminth parasites

#### 4.3.1 Trematoda/Neodermata/Digenea

*Fasciola hepatica* is an increasingly reported zoonotic foodborne pathogen in Asia, Africa, and South America. The parasite has a large genome that is highly polymorphic, permitting adaptation and invasion of a broad range of hosts (Lalor et al., 2021). The sequencing of *F. hepatica* isolates led the way to the first discovery of *Neorickettsia* endobacterium (nFh) in a prevalent human trematode pathogen (McNulty et al., 2017). *Neorickettsia* (Alphaproteobacteria, Rickettsiales/Anaplasmataceae) are obligate intracellular bacterial pathogens that were found to colonize some digenean trematodes. The bacteria can be transmitted from the infected trematode to the mammalian host tissues, invading and multiplying intracellularly, leading to a severe disease (Greiman et al., 2016; Fischer et al., 2017). The exact nature of *Neorickettsia*–digenean flukes association is not clear, but it is suggested that the bacteria are not essential for flukes’ survival (Formenti et al., 2020). In *F. hepatica*, nFh bacterium was detected in the female reproductive tract, Mehlis gland, eggs, seminal vesicle, and oral sucker. The location of the bacteria in reproductive tissues and eggs suggests a vertical propagation route, and their presence in the oral sucker that fixes the worm to the biliary epithelium may indicate a method of infection of the mammalian host (McNulty et al., 2017).

*Schistosoma mansoni* is an intravascular blood fluke, which is the cause of intestinal schistosomiasis, a common neglected tropical parasitic disease (Mawa et al., 2021). Worldwide, approximately 236.6 million people have schistosomiasis, mostly attributed to *S. mansoni*. The parasite is found in Africa, Middle East, the Caribbean, Brazil, Venezuela, and Suriname. In spite of the large-scale mass drug administration programs using praziquantel as preventive chemotherapy since 2003, schistosomiasis continues to be prevalent (Kittur et al., 2019; Levecke et al., 2020). Mawa et al. (2021) elucidated that schistosomes are highly complex multihost parasitic organisms, for which several features of the biology, virulence, worm–host interactions, and epidemiology remain largely unknown. Formenti et al. (2023) explored *S. mansoni* microbiome throughout the fluke life cycle stages, using fluorescence *in situ* hybridization employing a pan bacterial 16S rRNA gene-specific probe. The research group reported bacteria in the gut and tegument of adult worms, the apical and lateral glands of the eggs and miracidia, and the oral and ventral suckers of the cercariae, which required further characterization of the harbored bacteria. Similarly, a previous study has demonstrated the colonization of the tegument and gastrodermis of adult *S. japonicum* by bacterial communities (Gobert et al., 2022). Formenti et al. (2020) clarified that worm core microbiome is an intrinsic part of the parasite and affects its biology, especially if associated with worm gonads, and is vertically transmitted to the next progeny, and is therefore detected across parasite life cycle stages. Alternatively, transiently harbored bacteria coating the surface of free-living larval stages or carried within the alimentary tract of the parasite do not seem to affect the parasite biology and its biochemical pathways but perhaps the disease it produced. *Opisthorchis viverrini* juveniles in the duodenum of the infected host have been demonstrated to ingest *Helicobacter pylori* and to carry the bacilli to the bile ducts where the flukes reside. The combined action of the parasite and bacillus has been suggested in the etiology of opisthorchiasis-associated cholangiocarcinoma (Deenonpoe et al., 2017).

#### 4.3.2 Cestoda/Neodermata/Eucestoda

Cestodes (tapeworms) are distinctive among all helminths as they are perfectly specialized for the parasitic life (Tsai et al., 2013). The two most prevalent cestodes, *Echinococcus* and *Taenia*, present a major challenge to public health in various communities. Larval cestode infections are difficult to treat, with only few available antiparasitic chemotherapeutics (Hemphill et al., 2014). Research on these tapeworms remains limited, and the associated diseases have been added to the list of neglected tropical diseases (Mahanty, 2016; Ríos-Valencia et al., 2023). Tapeworms are defective in the piRNA pathway, one of the key mechanisms for silencing mobile genetic elements (MGE), which is important for preserving genome integrity between generations (Skinner et al., 2014). Herz and Brehm (2019) are the first to demonstrate the integration of densoviruses (ssDNA viruses) classified as Parvoviridae into the genome of different parasitic cestodes (*Echinococcus* spp., *Taenia* spp., and *Hymenolepis* spp.). The authors reported that further investigation into virus–cestode endogenization can assist in clearing up MGE silencing mechanisms in tapeworms. Dheilly et al. (2022) revealed an extensive diversity of viruses in parasitic Neodermata worms and reported that their role in flatworm-associated diseases must be focused on.

## 5 Endosymbionts of parasitic arthropods

*Sarcoptes scabiei* (Arachnida/Acariformes/Astigmata) is a parasitic mite that is the causative agent of an important human skin infectious disease—scabies. It is estimated that more than 300 million people are infected each year (Martin et al., 2018). The association between the mite infestation and secondary bacterial infections is increasingly reported and can lead to serious complications, including life-threatening *Staphylococcus aureus* bacteremia and severe post-streptococcal sequelae (Bowen et al., 2014; Thornley et al., 2018). Treatment and control of scabies are still arduous, as the available acaricides do not kill the eggs, which is the crucial life cycle stage for infection establishment (Wang et al., 2022). Swe et al. (2019) employed metagenome sequencing to clarify the internal microbial composition of *S. scabiei* adult females and eggs. *Streptomyces* spp., a Gram-positive, multicellular, spore-forming bacteria, was identified in both mite stages, with higher density in the adult female, especially in the gut. The authors reported that the bacteria may have an essential symbiotic role in the mite gut and may provide it with a survival advantage.

*Demodex folliculorum* (Arachnida/Acariformes/Prostigmata) is a mite that inhabits the pilosebaceous unit of the human skin. *D. folliculorum* and *D. brevis*, which are the most complex of the human skin microbiome, dwell mainly as commensals but can also trigger skin diseases such as rosacea, pityriasis folliculorum, and blepharitis (Forton, 2020; Wei et al., 2024). A number of *Bacillus* spp. (*oleronius, cereus*, and *simplex*), mainly Gram-positive bacteria, have been recorded to be associated with *D. folliculorum*, particularly the exoskeleton (Lacey et al., 2007; Tatu et al., 2017; Kubiak et al., 2018). Clanner‐Engelshofen et al. (2020) described, for the first time, the Gram-positive bacterium *Corynebacterium kroppenstedtii demodicis* as the endosymbiont of *D. folliculorum* mites. They reported that the bacteria that help the mite in the digestion of the lipid-rich sebum can be vertically transmitted to the progeny and appear to be essential for its survival.

*Pediculus humanus* (Insecta/Phthiraptera/Anoplura) is a hematophagous ectoparasite intimately connected to its human host. Two ecotypes of *Pediculus* infest humans: *P. humanus humanus*, the body louse, and *P. humanus capitis*, the head louse. *Pediculus* has been classified into six divergent mitochondrial clades from A to E, with clade A being the most prevalent. The head louse encloses the full genetic diversity of the clades, while the body louse belongs to clades A and D (Amanzougaghene et al., 2019). Body lice are known to transmit bacterial pathogens, mainly *Rickettsia prowazekii*, *Borrelia recurrentis*, and *B. quintana*, while the vector capacity of *P. h. capitis* is still subject to debate (Feldmeier, 2023). *Acinetobacter* spp., a Gram-negative gamma-proteobacteria, have been detected with high prevalence in head louse, suggesting its potential role for transmission of the bacteria (Sunantaraporn et al., 2015). *P. humanus* harbors a primary endosymbiotic bacterium *Candidatus Reisia pediculicola*, which is a Gram-negative gamma-proteobacterium that is transovarially transmitted to the progeny and located in the mycetome on the ventral side of the midgut (Kirkness et al., 2010). The bacteria are fundamental for lice development and survival, complementing their unbalanced diet and synthesizing missing elements (Hammoud et al., 2022). Agany et al. (2020) described the microbiome differences between the head and body lice. They reported a higher load of vertically transmitted bacterial endosymbiont in the head louse and distinct harbored non-endosymbiotic bacterial groups between the two ecotypes. The endosymbiotic bacteria have been proposed as a novel target for lice control, since control measures are challenged by the recent spread of insecticide resistance alleles (Hammoud et al., 2022).

*Wohlfahrtia magnifica* (Insecta/Diptera/Sarcophagidae) is an obligate parasite, as the fly is dependent on a vertebrate host to complete its life cycle. It is an important cause of myiasis in animals and humans (Schröttner et al., 2017; Wakid et al., 2022). Cutaneous myiasis usually occurs accidentally in humans through the infestation of non-healing wounds in high-risk groups by parasitic larvae. Bacteria have been isolated from the different developmental stages and larval organs of *W. magnifica* and were later identified as *Wohlfahrtiimonas chitiniclastica* and *Ignatzscheneria* spp., which are Gram-negative rods classified as Gammaproteobacteria (Toth et al., 2008; Reed et al., 2021). The relationship between the fly and the hosted bacteria may be symbiotic, as the bacteria are suggested to play a role in fly metamorphosis due to their strong chitinase activity. These bacteria have been detected in other parasitic flies (Schröttner et al., 2017; Maleki-Ravasan et al., 2020) as well. *Wohlfahrtimonas* and *Ignatzscheneria* spp. have been associated with rare human infections and sepsis, typically related to wound infestation by maggots of flesh flies (Connelly et al., 2019; Ahmad et al., 2022; Kozyk et al., 2023).

## 6 Discussion

### 6.1 Comparative analysis of immune modulation by endosymbionts and clinical implications

The tripartite relation of host–parasite–endosymbiont is multidimensional, where the human is considered the macro-host and the parasite is the micro-host. Since the existence of the endosymbiont can be directly linked to its micro-host, their survival can be mutually dependent, and the endosymbiont may influence the macro-host immune response in favor of parasite persistence. This work presents an overview from data collected regarding endosymbiosis in more than 20 species of parasites of medical importance (**Table 1**) to understand the endosymbiont-driven immunomodulatory effects.

**Table 1:** Summary of findings across various parasite–endosymbiont systems.

Parasite–endosymbiont system		Type	Evidence	Possible transmission mode	Biological/Immunological relevance	References
*Acanthamoeba* spp.	*L. pneumophila, Aeromonas, E. coli, Shigella, P. aeruginosa, V. cholerae, Mycobacteria, Chlamydia, Rickettsia, Coxiella, Bartonella*	Bacteria	Reported as *Acanthamoeba*-associated bacteria through molecular detection	Environmental	-Amoeba proposed as reservoir and protective niche from environmental and host immune stresses - Potential enhancement of bacterial persistence/pathogenicity	Siddiqui and Khan, 2012; Mungroo et al., 2021; Rayamajhee et al., 2022; Price et al., 2024
	Giant viruses/Mimiviridae APMV	Giant dsDNA viruses	Multiple giant viruses identified in *Acanthamoeba* through metagenomic analysis	Environmental (phagocytosis)	-Possible effects on amoeba biology, inhibits serine proteinase expression, and affects encystation	Colson et al., 2013, 2017; Boratto et al., 2015; Queiroz et al., 2022
*Entamoeba* spp.	VLPs	Virus/VLP	VLPs observed by electron microscopy of *in vitro* culture	Vertical (cytoplasmic)	-Possible parasite lysis; identity and significance unresolved	Diamond et al., 1972; Hruska et al., 1974; Mattern et al., 1977
	Naryaviridae Nenyaviridae	CRESS virus (ssDNA)	Metagenomic analysis	Unknown	-Significance unresolved	Kinsella et al., 2020
	Redondoviridae	CRESS virus (ssDNA)	Metagenomic analysis	Unknown	-Amoeba proposed as replication niche -Possible association with oral/respiratory disease	Keeler et al., 2023
*Leishmania* spp.	LRV1/LRV2	dsRNA virus	Naturally occurring *Leishmania* viruses observed by electron microscopy, exosome pathway analysis, molecular analysis	Vertical (cytoplasmic), exosome-mediated horizontal transfer	-TLR3 activation, hyperinflammation, increased parasite burden/persistence - Increased GP63/MPI expression, immune evasion, and disease exacerbation	Eren et al., 2016; Ginouvès et al., 2016; Hartley et al., 2016; Atyade et al., 2019; Saberi et al., 2019; Mirabedini et al., 2023; Sabeti et al., 2025
	LmarLBV1	Negative-sense ssRNA virus	Identified by sequencing in *L. martiniquensis*	Vertical (cytoplasmic)	-Significance unresolved	Grybchuk et al., 2020
*Trypanosoma cruzi*	Enveloped/non-enveloped VLPs	Unknown	VLP clusters observed in epimastigotes by electron microscopy	Unknown (possibly acquired from the vector)?	-Unknown	Fernández-Presas et al., 2017
*Trichomonas vaginalis*	TVV1–TVV4	dsRNA virus	TVV species identified by electron microscopy, molecular analysis, ECVs analysis	Vertical (cytoplasmic), exosome-mediated horizontal transfer	-Associated with disease severity -Increased immunogenic proteins and virulence-associated phenotypes - TVV-containing sECVs induce stronger proinflammatory responses	Benchimol et al., 2002; Fichorova et al., 2013; 2017; Graves et al., 2019a, 2019b; Rada et al., 2022
	TVV5	dsRNA virus	New species identified by transcriptome mining	Unknown	Unknown	Manny et al., 2022
	*Mycoplasma hominis* *Ca. Mycoplasma girerdii*	Bacteria	Frequent intracellular association observed by electron microscopy, *in vitro* mechanistic studies	Horizontal from infected parasite to another	-Arginine competition, altered NO response -Increased cytoadherence/hemolysis and inflammation	Margarita et al., 2016; Fichorova et al., 2017; Dessì et al., 2019; de Aquino and Simoes-Barbosa, 2022
*Giardia lamblia*	Giardiavirus (GLV) Vilyaviridae	dsRNA virus CRESS virus (ssDNA)	Electron microscopy, metagenomic analysis	Unknown	-High viral titers can arrest parasite growth	Garlapati and Wang, 2004; Janssen et al., 2015; Kinsella et al., 2020; Marucci et al., 2021
*Cryptosporidium*	CspV1	dsRNA virus	Molecular sequencing/subtyping, sequence divergence among viruses associated with *C. parvum*, *C. hominis*, *C. felis*, and *C. meleagridis*	Vertical (during cell division)	-Increased fecundity -Parasite immune evasion through Type-I IFN activation and attenuation of IFN-γ-mediated epithelial defense	Jenkins et al., 2008, 2015; Adjou et al., 2023; Deng et al., 2023; Graham et al., 2023
*Toxoplasma gondii*	*Apocryptovirus odysseus* (Ao)	ssRNA narnavirus	Computational association with 2 hypervirulent strains	Unknown	-Predicted proinflammatory activity -Proposed hypervirulence factor	Gupta et al., 2024
*Plasmodium vivax*	MaRNAV-1	ssRNA narnavirus-like virus	Molecular analysis	Unknown (possibly acquired from the vector)?	Unknown	Charon et al., 2019; Petrone et al., 2026
*Blastocystis* spp.	Putative bacteria VLPs	-----------	Limited historical observations	------------------	----------------------	Shaw et al., 2026
Onchocercid filariae	*Wolbachia*	Bacteria	Extensive molecular, anatomical, and functional evidence	Vertical (transovarial)	-Parasite development, fertility and viability -Th1-associated inflammation and contribution to pathology	Taylor et al., 2005; Turner et al., 2006; Ferri et al., 2011; Hübner et al., 2022; Setegn et al., 2024
*Ascaris lumbricoides*	Gut microbiome	Bacterial community	Metagenomic studies demonstrate a distinct worm microbiome and burden-associated differences	Unknown (possibly environmental)	Unknown	Klomkliew et al., 2022; Muslim et al., 2024
*Fasciola hepatica*	Neorickettsia nFh	Bacteria	Genome sequencing and localization in reproductive tissues, eggs, and oral sucker	Vertical (possibly transovarial)	Unknown	McNulty et al., 2017; Greiman et al., 2016; Fischer et al., 2017; Formenti et al., 2020
*Schistosoma* spp.	Bacterial microbiome	Bacterial community	Genome sequencing and localization in gut, tegument, and multiple life-cycle stages	Unknown (possibly environmental)	Unknown	Formenti et al., 2020; Gobert et al., 2022; Formenti et al., 2023
*Opisthorchis viverrini*	*Helicobacter pylori*	Bacteria	Molecular detection in human stool samples to detect association	Possibly juvenile flukes ingest and carry *H. pylori* toward bile ducts	-Proposed combined parasite–bacteria interaction in cholangiocarcinoma etiology	Deenonpoe et al., 2017
Cestodes (*Echinococcus*, *Taenia*, *Hymenolepis*)	Densovirus-derived sequences	ssDNA virus/endogenous viral elements	Metagenomics	Vertical (viral sequences integrated into cestode genomes)	Potential relevance to MGE/piRNA biology	Herz and Brehm, 2019
*Sarcoptes scabiei*	*Streptomyces* spp.	Bacteria	Metagenomic detection in adult females’ gut and eggs	Unknown (possibly environmental)	-Possible parasite survival advantage	Swe et al., 2019
*Demodex folliculorum*	*Bacillus* spp.	Bacteria	Culture and mass spectrometry	Unknown	-Possibly associated with skin disease	Lacey et al., 2007; Tatu et al., 2017; Kubiak et al., 2018
	*Corynebacterium kroppenstedtii demodicis*	Bacteria	Molecular analysis	Proposed vertical transmission	-Lipid digestion and mite survival	Clanner-Engelshofen et al., 2020
*Pediculus humanus*	*Candidatus Reisia pediculicola*	Bacteria	Localized in mycetome, identified by genome sequencing and phylogenetics	Vertical (transovarial)	-Essential nutritional/developmental function	Kirkness et al., 2010; Agany et al., 2020; Hammoud et al., 2022
	*Acinetobacter* spp.	Bacteria	Molecular analysis		-Possible bacterial transmission role	Sunantaraporn et al., 2015; Agany et al., 2020
*Wohlfahrtia magnifica*	*Wohlfahrtiimonas chitiniclastica, Ignatzscheneria* spp.	Bacteria	Isolated from fly developmental stages by culture and molecular analysis	Unknown (possibly environmental)	-Strong chitinase activity suggesting role in metamorphosis -Possible association with wound myiasis in humans	Toth et al., 2008; Schröttner et al., 2017; Reed et al., 2021; Connelly et al., 2019; Maleki-Ravasan et al., 2020

Across the parasite–endosymbiont systems reviewed above, only three systems were extensively studied, involving *Leishmania*, *Trichomonas*, and filarial nematodes. The *Leishmania*–LRV and *Trichomonas*–TVV demonstrate a common theme; both exacerbate the proinflammatory response, which can lead to host-damaging hyperinflammation and, at the same time, allow evasion and persistence. These dsRNA viral endosymbionts provide clear examples of endosymbiont-mediated modulation of immune response and disease phenotype. In patients infected with *L. guyanensis* and suffering from American tegumentary leishmaniasis, LRV-1 was shown to be associated with higher levels of intralesional inflammatory markers, resistance to pentamidine therapy, and relapsing symptoms (Bourreau et al., 2016). In parallel research including patients infected with *L. guyanensis*, LRV-1 was linked to higher levels of the pro-inflammatory cytokine interleukin-17A (IL-17A) and lower levels of the protective IFN-γ. In addition, chronic disease was observed more frequently among LRV1-positive patients than among LRV1-negative patients. Furthermore, in murine models, LRV1-associated *Leishmania* infection resulted in metastatic lesions in the presence of IL-17A and in the absence of IFN-γ (Hartley et al., 2016). A similar interaction was reported in *T. vaginalis*, with exaggerated symptomatology in infected females in the presence of TVV. However, the clinical spectrum observed showed variability and inconsistency between the different studies, probably due to the small sample sizes included (Fichorova et al., 2017). *In vitro* models demonstrated the TVV-driven robust inflammatory response mediated by the increased expression of IL-8 and RANTES, especially in the presence of pathogenic vaginal bacteria rather than the commensal *Lactobacillus*. Notably, parasite treatment with metronidazole was reported to increase the release of TVV, thus enhancing the inflammatory response, highlighting the effect of treatment on endosymbiont-associated immunopathology (Fichorova et al., 2012; 2013).

The second theme is the endosymbiont bacteria as the primary immunogen in the filaria–*Wolbachia* system. Remarkably, among all the endosymbionts described in parasites, *Wolbachia* is the one clearly established example of obligate mutual stable endosymbiont indispensable for parasite survival. *Wolbachia* is recently regarded as the dominant culprit in filariasis, not the worm itself; it stimulates a potent inflammatory and TH-1 immune response, resulting in disease pathogenesis and tissue damage. Similar to *T. vaginalis*–TVV, antiparasitic treatment and death of worms is associated with the release of *Wolbachia* into the blood, inducing systemic hyperinflammation (Turner et al., 2006; Setegn et al., 2024). The third theme is the endosymbiont as an immunologically silent partner, which can be revealed in the ectoparasite–endosymbiont systems, namely, the *P. humanus*–*Riesia* system, the *S. scabiei*–*Streptomyces* system, and the *D. folliculorum*–*Corynebacterium* system. These bacterial endosymbionts are described to play a primary role in parasite nutrition and development without evident immunological consequences. In contrast, immune response and disease complications that may arise are indirectly caused by the secondary bacterial infections rather than the endosymbiont (Kirkness et al., 2010; Swe et al., 2019; Clanner-Engelshofen et al., 2020). The fourth theme is the parasite as a replicative reservoir for the endosymbiont, for which the *Acanthamoeba* is the best example as it provides a protective niche for the microorganisms harbored within it—such as *Legionella*, *Mycobacteria*, and *Chlamydia*—and furthermore allows selection and evolution of virulent and immune evasion traits before exposure to the macro-host. A final note, Acanthamoeba-APMV interaction, in which the virus suppresses the expression of encystation-associated serine proteinase shares a broader functional similarity with the Leishmania–LRV interaction, in which the virus promotes infected macrophage survival through phosphorylation of AKT1; these interactions illustrate how virus-associated processes can manipulate the hosting cell (Boratto et al., 2015; Queiroz et al., 2022; Price et al., 2024).

### 6.2 Therapeutic opportunities

Obligate mutual symbiotic relations are mainly based on metabolic complementation and biochemical pathway potentiation in one or both hosts (Manoj et al., 2021). This obligate dependence of parasites on their endosymbionts offers therapeutic opportunities either by directly targeting the endosymbiont, by altering host pathways in parasite–symbiont interaction, or by exploiting endosymbiotic virus for efficient drug delivery. Distinguishing between transient microbial invaders, organisms sheltering inside more resilient parasites, and true endosymbionts is a pivotal step in understanding immunomodulatory impacts and therapeutic targets. Differentiating criteria involve several aspects such as location of microorganism within the parasite, transmission mode, stability of the association, genome characteristics, and functional dependence; these aspects are still unresolved in the majority of parasite–endosymbionts systems. The Parasite Microbiome Project highlighted the challenges in parasite endosymbiosis research including low-biomass microbial nucleic acid recovery and emphasized the need for standardized approach and metagenomic protocol to identify true endosymbionts from transient environmental microbes (Dheilly et al., 2019).

The strongest proof of concept is the filaria–*Wolbachia* system where endosymbiont targeting efficiently curtails the pathogenicity of the parasite. *Wolbachia* is vertically transmitted, stable, and indispensable for the survival of its filarial nematode host. Molecular studies have revealed that parasites such as *B. malayi* and *O. volvulus* have lost the ability to synthesize important biomolecules including heme, nucleotides, riboflavin, and certain lipids. This extensive metabolic interdependence creates a therapeutic opportunity, making their biosynthesis pathways potential targets for anti-*Wolbachia* drug design, thus impairing the parasite viability and development (Slatko et al., 2010). Doxycycline is established as an effective and safe anti-*Wolbachia* drug, is recommended as a treatment option for human filarial infections, is given as a prolonged regimen (ranging from 4 to 6 weeks), and is contraindicated in children and pregnant women. Therefore, doxycycline is typically prescribed for individual cases but not for mass drug administration. In their comprehensive review, Risch et al. (2024) discussed potential antifilarial drugs in development, including anti-*Wolbachia* candidates flubentylosin—a macrolide antibiotic, AWZ1066S compound, and rifampicin. The trials on the first two candidates were discontinued for weak efficacy and safety concerns, respectively, while rifampicin studies are ongoing, but its wide use can negatively impact its effect as an anti-tuberculous agent.

In addition to the well-established filaria–*Wolbachia* model for taking advantage of the parasite–endosymbiont relation in anti-parasitic therapeutics, the study of the potential value of viral endosymbionts to combat their hosting parasite has gained increasing attention, but remain largely experimental. The exploitation of the cytopathic effect of viruses against their sheltering host is a strategy under active investigation. The *G. lamblia* GLV virus stands out as the most promising candidate due to the inhibitory effect of increased viral load on parasite growth. Li et al. (2025) demonstrated that GLV can alleviate intestinal pathology in *G. lamblia*-infected mice, where GLV was found to disperse into the extracellular space to infect *G. lamblia* trophozoites by hijacking the parasites’ own vesicular system. The more selective the virus is to its host, and the simpler the life cycle of the parasite facilitating coordinated multiplication peaks in both agents, the more promising is the viral agent as a potential pharmaceutical tool in parasite control (Barrow et al., 2020). Another tactic in the use of viral endosymbionts in antiparasitic management involves exploiting VLPs. Candidate therapeutic agents can be delivered either inside the empty viral protein shells or attached to the surface of VLPs. This approach has provided promising results in the field of plant parasitology and is still under investigation for medically important parasites (Cao et al., 2015). VLPs can also be used as molecular vehicles to deliver genetic tools that can block the translation of essential parasite genes involved in vital metabolic processes (Zhang et al., 2018; Dan et al., 2000). Antisense sequences, DNA oligomers modified with methylenemorpholine rings (morpholinos), hammerhead ribozymes, and CRISPR-Cas are all promising techniques that were shown to induce at least transient modulation of parasite gene expression (Barrow et al, 2020). Finally, host-directed approaches are an important strategy in targeting components of the host immune system activated by the parasite–endosymbiont interactions. Viral agents hijack components of the host immune system to favor parasite survival for their own benefit. TLR3, type I interferon signaling, and pathways implicated in pro-inflammatory cytokine release are all potential therapeutic targets to dampen immunopathology associated with parasitic infections (Barrow et al., 2020**;** Mirabedini et al., 2023).

## 7 Conclusion

The endosymbiotic relationships documented across the presented array of medically important parasites revealed a diversity of microbial partners that, in many instances, shape parasite fitness and virulence, and consequently impact the human host immune response to the parasitic infection and the natural history of the disease. This review examined nearly 20 parasite–endosymbiont systems, focusing on their impact on host–parasite interactions and highlighting their potential as therapeutic targets. Within this context, the filaria–*Wolbachia*, *Leishmania*–LRV, and *Trichomonas*–TVV models demonstrate a significant proof that endosymbionts can directly drive immunomodulation in the human host, such as innate immune hyperresponsiveness, inflammatory cascades, and cytokine dysregulation, thus worsening the clinical outcome. Understanding these relationships introduced the concept of endosymbiotic-directed treatment, which became concrete in *Wolbachia-*targeted therapy in LF and onchocerciasis. Besides the bacterial endosymbiont-directed treatment, exploiting the viral endosymbiont and virus-derived delivery systems for the biological control of the protozoal host is an emerging therapeutic approach under investigation. Amid this landscape, the *Acanthamoeba*–endosymbionts model stands out as a hub for a remarkable diversity of bacteria that can cause disease independent of the parasite. This characterizes *Acanthamoeba* as a reservoir and environmental training ground that allows bacterial virulence evolution. Reciprocally, the bacteria influence *Acanthamoeba* encystment and pathogenic potential. Finally, it is worth mentioning that the depth of characterization of endosymbionts across parasite taxa is markedly uneven. Extensively studied systems such as filaria–*Wolbachia* and *Leishmania*–LRV contrast sharply with other parasites such as *Blastocystis*, *Sarcoptes*, *Schistosoma*, and the cestodes, where microbiome data remain limited, fragmentary, and inconclusive.

Disclosing the world of microorganisms living within parasites, especially with the advanced metagenomic analysis, represents a research frontier of immense biomedical significance. It can provide fundamental data concerning parasite biology, host–parasite interactions, and vulnerabilities that could be targeted for efficient treatment and control measures.

## References

[B1] Abbas A. A. Taylor L. J. Dothard M. I. Leiby J. S. Fitzgerald A. S. Khatib L. A. . (2019). Redondoviridae, a family of small, circular DNA viruses of the human oro-respiratory tract associated with periodontitis and critical illness. Cell Host Microbe 25, 719–729. doi: 10.1016/j.chom.2019.04.001 31071295 PMC6510254

[B2] Adam R. D. (2021). *Giardia duodenalis*: biology and pathogenesis. Clin. Microbiol. Rev. 34, e00024-19. doi: 10.1128/CMR.00024-19 34378955 PMC8404698

[B3] Adjou K. T. Chevillot A. Lucas P. Blanchard Y. Louifi H. Arab R. . (2023). First identification of *Cryptosporidium parvum* virus 1 (CSpV1) in various subtypes of *Cryptosporidium parvum* from diarrheic calves, lambs and goat kids from France. Vet. Res. 54, 66. doi: 10.1186/s13567-023-01196-4 37608341 PMC10464362

[B4] Afzelius B. A. Alberti G. Dallai R. Godula J. Witalinski W. (1989). Virus-and Rickettsia-infected sperm cells in arthropods. J. Invertebr. Pathol. 53, 365–377. doi: 10.1016/0022-2011(89)90102-x

[B5] Agany D. D. Potts R. Hernandez J. L. G. Gnimpieba E. Z. Pietri J. E. (2020). Microbiome differences between human head and body lice ecotypes revealed by 16S RRNA gene amplicon sequencing. J. Parasitol. 106, 14–24. doi: 10.1645/19-132 31958374

[B6] Ahmad Y. Gaston D. C. Gray J. Zhong D. Gudenkauf B. Bourdas D. . (2022). The brief Case: the Fly who cried Wohlf. J. Clin. Microbiol. 60, e01073-21. doi: 10.1128/jcm.01073-21 35703540 PMC9199405

[B7] Alves J. M. Klein C. C. da Silva F. M. Costa-Martins A. G. Serrano M. G. Buck G. A. . (2013). Endosymbiosis in trypanosomatids: the genomic cooperation between bacterium and host in the synthesis of essential amino acids is heavily influenced by multiple horizontal gene transfers. BMC Evol. Biol. 13, 190. doi: 10.1186/1471-2148-13-190 24015778 PMC3846528

[B8] Amanzougaghene N. Fenollar F. Davoust B. Djossou F. Ashfaq M. Bitam I. . (2019). Mitochondrial diversity and phylogeographic analysis of *Pediculus humanus* reveals a new Amazonian clade “F. Infect. Genet. Evol. 70, 1–8. doi: 10.1016/j.meegid.2019.02.006 30769089

[B9] Atayde V. D. Filho A. Chaparro V. Zimmermann A. Martel C. Jaramillo M. . (2019). Exploitation of the *Leishmania* exosomal pathway by *Leishmania* RNA virus 1. Nat. Microbiol. 4, 714–723. doi: 10.1038/s41564-018-0352-y 30692670

[B10] Barrow P. Dujardin J. C. Fasel N. Greenwood A. D. Osterrieder K. Lomonossoff G. . (2020). Viruses of protozoan parasites and viral therapy: Is the time now right? Virol. J. 17, 142. doi: 10.1186/s12985-020-01410-1 32993724 PMC7522927

[B11] Bastilho A. L. Brito R. M. Andrade J. L. da Rocha Rihs J. B. de Brito Duval I. Cardozo M. E. . (2025). *Toxoplasma gondii* non-archetypal strain induces lung inflammation during acute and early chronic infection in mice. Parasitology 152, 497–509. doi: 10.1017/S0031182025000538 40223760 PMC12278009

[B12] Benchimol M. Monteiro S. P. Chang T. H. Alderete J. F. (2002). Virus in *Trichomonas*—an ultrastructural study. Parasitol. Int. 51, 293–298. doi: 10.1016/s1383-5769(02)00016-8 12243783

[B13] Boratto P. Albarnaz J. D. Almeida G. M. Botelho L. Fontes A. C. Costa A. O. . (2015). *Acanthamoeba polyphaga* mimivirus prevents amoebal encystment-mediating serine proteinase expression and circumvents cell encystment. J. Virol. 89, 2962–2965. doi: 10.1128/JVI.03177-14 25520511 PMC4325711

[B14] Bouchery T. Lefoulon E. Karadjian G. Nieguitsila A. Martin C. (2013). The symbiotic role of *Wolbachia* in Onchocercidae and its impact on filariasis. Clin. Microbiol. Infect. 19, 131–140. doi: 10.1111/1469-0691.12069 23398406

[B15] Bourreau E. Ginouves M. Prévot G. Hartley M. A. Gangneux J. P. Robert-Gangneux F. . (2016). Presence of Leishmania RNA virus 1 in *Leishmania guyanensis* increases the risk of first-line treatment failure and symptomatic relapse. J. Infect. Dis. 213, 105–111. doi: 10.1093/infdis/jiv355 26123564

[B16] Bousbia S. Papazian L. Saux P. Forel J. M. Auffray J. P. Martin C. . (2013). Serologic prevalence of amoeba-associated microorganisms in intensive care unit pneumonia patients. PloS One 8, e58111. doi: 10.1371/journal.pone.0058111 23469263 PMC3585915

[B17] Bowen A. C. Tong S. Y. Chatfield M. D. Carapetis J. R. (2014). The microbiology of impetigo in indigenous children: associations between *Streptococcus pyogenes*, *Staphylococcus aureus*, scabies, and nasal carriage. BMC Infect. Dis. 14, 727. doi: 10.1186/s12879-014-0727-5 25551178 PMC4299569

[B18] Caccio S. M. Lalle M. Svärd S. G. (2018). Host specificity in the *Giardia duodenalis* species complex. Infect. Genet. Evol. 66, 335–345. doi: 10.1016/j.meegid.2017.12.001 29225147

[B19] Cantanhede L. M. Fernandes F. G. Ferreira G. E. Porrozzi R. Ferreira R. D. Cupolillo E. (2018). New insights into the genetic diversity of *Leishmania* RNA Virus 1 and its species-specific relationship with *Leishmania* parasites. PloS One 13, e0198727. doi: 10.1371/journal.pone.0198727 29912912 PMC6005476

[B20] Cao J. Guenther R. H. Sit T. L. Lommel S. A. Opperman C. H. Willoughby J. A. (2015). Development of abamectin loaded plant virus nanoparticles for efficacious plant parasitic nematode control. ACS Appl. Mate Interfaces 7, 9546–9553. doi: 10.1021/acsami.5b00940 25906360

[B21] Casiraghi M. Bordenstein S. R. Baldo L. Lo N. Beninati T. Wernegreen J. J. . (2005). Phylogeny of *Wolbachia pipientis* based on gltA, groEL and ftsZ gene sequences: clustering of arthropod and nematode symbionts in the F supergroup, and evidence for further diversity in the *Wolbachia* tree. Microbiology 151, 4015–4022. doi: 10.1099/mic.0.28313-0 16339946

[B22] Charon J. Grigg M. J. Eden J. S. Piera K. A. Rana H. William T. . (2019). Novel RNA viruses associated with *Plasmodium vivax* in human malaria and Leucocytozoon parasites in avian disease. PloS Pathog. 15, e1008216. doi: 10.1371/journal.ppat.1008216 31887217 PMC6953888

[B23] Clanner‐Engelshofen B. M. French L. E. Reinholz M. (2020). *Corynebacterium kroppenstedtii* subsp. demodicis is the endobacterium of *Demodex folliculorum*. J. Eur. Acad. Dermatol. Venereol. 34, 1043–1049. doi: 10.1111/jdv.16069 31713895

[B24] Colson P. De Lamballerie X. Yutin N. Asgari S. Bigot Y. Bideshi D. K. . (2013). Megavirales”, a proposed new order for eukaryotic nucleocytoplasmic large DNA viruses. Arch. Virol. 158, 2517–2521. doi: 10.1007/s00705-013-1768-6 23812617 PMC4066373

[B25] Colson P. La Scola B. Levasseur A. Caetano-Anollés G. Raoult D. (2017). Mimivirus: leading the way in the discovery of giant viruses of amoebae. Nat. Rev. Microbiol. 15, 243–254. doi: 10.1038/nrmicro.2016.197 28239153 PMC7096837

[B26] Connelly K. L. Freeman E. Smibert O. C. Lin B. (2019). *Wohlfahrtiimonas chitiniclastica* bloodstream infection due to a maggot-infested wound in a 54-year-old male. J. Glob. Infect. Dis. 11, 125–126. doi: 10.4103/jgid.jgid_58_18 31543656 PMC6733193

[B27] Dan M. Wang A. L. Wang C. C. (2000). Inhibition of pyruvate-ferredoxin oxidoreductase gene expression in Giardia lamblia by a virus-mediated hammerhead ribozyme. Mol. Microbiol. 36, 447–456. doi: 10.1046/j.1365-2958.2000.01863.x 10792730

[B28] de Almeida M. E. Koru O. Steurer F. Herwaldt B. L. da Silva A. J. (2017). Detection and differentiation of *Leishmania* spp. in clinical specimens by use of a SYBR green-based real-time PCR assay. J. Clin. Microbiol. 55, 281–290. doi: 10.1128/JCM.01764-16 27847378 PMC5228241

[B29] de Aquino M. F. Simoes-Barbosa A. (2022). A microbial piñata: bacterial endosymbionts of *Trichomonas vaginalis* come in different flavors. MBio 13, e01323-22. doi: 10.1128/mbio.01323-22 35968950 PMC9426529

[B30] Deenonpoe R. Mairiang E. Mairiang P. Pairojkul C. Chamgramol Y. Rinaldi G. . (2017). Elevated prevalence of *Helicobacter* species and virulence factors in opisthorchiasis and associated hepatobiliary disease. Sci. Rep. 7, 42744. doi: 10.1038/srep42744 28198451 PMC5309894

[B31] Deng S. He W. Gong A. Y. Li M. Wang Y. Xia Z. . (2023). *Cryptosporidium* uses CSpV1 to activate host type I interferon and attenuate antiparasitic defenses. Nat. Commun. 14, 1456. doi: 10.1038/s41467-023-37129-0 36928642 PMC10020566

[B32] Deng L. Tan K. S. (2025). From parasite to partner: unravelling the multifaceted role of Blastocystis in human health and disease. Lancet Microbe 6, 101155. doi: 10.1016/j.lanmic.2025.101155 40541220

[B33] Dessì D. Margarita V. Cocco A. R. Marongiu A. Fiori P. L. Rappelli P. (2019). *Trichomonas vaginalis* and *Mycoplasma hominis*: new tales of two old friends. Parasitology 146, 1150–1155. doi: 10.1017/S0031182018002135 30616707

[B34] Dheilly N. M. Lucas P. Blanchard Y. Rosario K. (2022). A world of viruses nested within parasites: unraveling viral diversity within parasitic flatworms (Platyhelminthes). Microbiol. Spectr. 10, e00138-22. doi: 10.1128/spectrum.00138-22 35536058 PMC9241645

[B35] Dheilly N. M. Martinez Martinez J. Rosario K. Brindley P. J. Fichorova R. N. Kaye J. Z. . (2019). Parasite microbiome project: Grand challenges. PloS Pathog. 15, e1008028. doi: 10.1371/journal.ppat.1008028 31600339 PMC6786532

[B36] Diamond L. S. Mattern C. F. Bartgis I. L. (1972). Viruses of *Entamoeba histolytica* I. Identification of transmissible virus-like agents. J. Virol. 9, 326–341. doi: 10.1128/JVI.9.2.326-341.1972 4335522 PMC356300

[B37] Eren R. O. Reverte M. Rossi M. Hartley M. A. Castiglioni P. Prevel F. . (2016). Mammalian innate immune response to a *Leishmania*-resident RNA virus increases macrophage survival to promote parasite persistence. Cell Host Microbe 20, 318–328. doi: 10.1016/j.chom.2016.08.001 27593513 PMC5493041

[B38] Feldmeier H. (2023). Head lice as vectors of pathogenic microorganisms. Trop. Med. Health 51, 53. doi: 10.1186/s41182-023-00545-5 37730694 PMC10510260

[B39] Fenn K. Blaxter M. (2004). Quantification of *Wolbachia* bacteria in *Brugia malayi* through the nematode lifecycle. Mol. Biochem. Parasitol. 137, 361–364. doi: 10.1016/j.molbiopara.2004.06.012 15383308

[B40] Fernández-Presas A. M. Padilla-Noriega L. Robert L. Jiménez J. A. Solano S. Delgado J. . (2017). Enveloped and non-enveloped viral-like particles in *Trypanosoma cruzi* epimastigotes. Rev. Inst. Med. Trop. S. Paulo 59, e46. doi: 10.1590/S1678-9946201759046 28793017 PMC5553942

[B41] Ferri E. Bain O. Barbuto M. Martin C. Lo N. Uni S. . (2011). New insights into the evolution of *Wolbachia* infections in filarial nematodes inferred from a large range of screened species. PloS One 6, e20843. doi: 10.1371/journal.pone.0020843 21731626 PMC3120775

[B42] Fichorova R. N. Buck O. R. Yamamoto H. S. Fashemi T. Dawood H. Y. Fashemi B. . (2013). The villain team-up or how *Trichomonas vaginalis* and bacterial vaginosis alter innate immunity in concert. Sex Transm. Infect. 89, 460–466. doi: 10.1136/sextrans-2013-051052 23903808 PMC3746192

[B43] Fichorova R. Fraga J. Rappelli P. Fiori P. L. (2017). *Trichomonas vaginalis* infection in symbiosis with Trichomonasvirus and *Mycoplasma*. Res. Microbiol. 168, 882–891. doi: 10.1016/j.resmic.2017.03.005 28366838 PMC8130574

[B44] Fichorova R. N. Lee Y. Yamamoto H. S. Takagi Y. Hayes G. R. Goodman R. P. . (2012). Endobiont viruses sensed by the human host–beyond conventional antiparasitic therapy. PloS One 7, e48418. doi: 10.1371/journal.pone.0048418 23144878 PMC3492353

[B45] Fischer K. Tkach V. V. Curtis K. C. Fischer P. U. (2017). Ultrastructure and localization of *Neorickettsia* in adult digenean trematodes provides novel insights into helminth-endobacteria interaction. Parasites Vectors 10, 177. doi: 10.1186/s13071-017-2123-7 28407790 PMC5390476

[B46] Formenti F. Cortés A. Brindley P. J. Cantacessi C. Rinaldi G. (2020). A bug’s life: delving into the challenges of helminth microbiome studies. PloS Negl.Trop. Dis. 14, e0008446. doi: 10.1371/journal.pntd.0008446 32911483 PMC7482834

[B47] Formenti F. Cortés A. Deiana M. Salter S. Parkhill J. Berriman M. . (2023). The human blood fluke, *Schistosoma mansoni*, harbors bacteria throughout the parasite's life cycle. J. Infect. Dis. 228, 1299–1303. doi: 10.1093/infdis/jiad288 37487539 PMC10629713

[B48] Forton F. M. (2020). The pathogenic role of *Demodex* mites in rosacea: a potential therapeutic target already in erythematotelangiectatic rosacea? Dermatol. Ther. 10, 1229–1253. doi: 10.1007/s13555-020-00458-9 33095403 PMC7649190

[B49] Garlapati S. Wang C. C. (2004). Identification of a novel internal ribosome entry site in Giardiavirus that extends to both sides of the initiation codon. J. Biol. Chem. 279, 3389–3397. doi: 10.1074/jbc.M307565200 14615487

[B50] Gerace E. Presti V. D. Biondo C. (2019). *Cryptosporidium* infection: epidemiology, pathogenesis, and differential diagnosis. Eur. J. Microbiol. Immunol. 9, 119–123. doi: 10.1556/1886.2019.00019 31934363 PMC6945992

[B51] Ginouvès M. Simon S. Bourreau E. Lacoste V. Ronet C. Couppié P. . (2016). Prevalence and distribution of *Leishmania* RNA virus 1 in *Leishmania* parasites from French Guiana. Am. J. Trop. Med. Hyg. 94, 102. doi: 10.4269/ajtmh.15-0419 26598572 PMC4710412

[B52] Gobert G. N. McManus D. P. McMullan G. Creevey C. J. Carson J. Jones M. K. . (2022). Adult schistosomes have an epithelial bacterial population distinct from the surrounding mammalian host blood. PloS One 17, e0263188. doi: 10.1371/journal.pone.0263188 35085360 PMC8794206

[B53] Goh E. J. H. Putera I. La Distia Nora R. Mahendradas P. Biswas J. Chee S. P. . (2023). Ocular toxoplasmosis. Ocul. Immunol. Inflamm. 31, 1342–1361. doi: 10.1080/09273948.2022.2117705 36095008

[B54] Graham M. L. Li M. Gong A. Y. Deng S. Jin K. Wang S. . (2023). *Cryptosporidium parvum* hijacks a host’s long noncoding RNA U90926 to evade intestinal epithelial cell-autonomous antiparasitic defense. Front. Immunol. 14, 1205468. doi: 10.3389/fimmu.2023.1205468 37346046 PMC10280636

[B55] Graves K. Ghosh A. P. Kissinger P. J. Muzny C. A. (2019a). *Trichomonas vaginalis* virus: a review of the literature. Int. J. STD AIDS 30, 496–504. doi: 10.1177/0956462418809767 30626281

[B56] Graves K. J. Ghosh A. P. Schmidt N. Augostini P. Secor W. E. Schwebke J. R. . (2019b). *Trichomonas vaginalis* virus among women with trichomoniasis and associations with demographics, clinical outcomes, and metronidazole resistance. Clin. Infect. Dis. 69, 2170–2176. doi: 10.1093/cid/ciz146 30768180 PMC6880324

[B57] Graves K. J. Novak J. Muzny C. A. (2025). *Trichomonas vaginalis* virus: current insights and emerging perspectives. Viruses 17, 898. doi: 10.3390/v17070898 40733518 PMC12298157

[B58] Greiman S. E. Rikihisa Y. Cain J. Vaughan J. A. Tkach V. V. (2016). Germs within worms: localization of *Neorickettsia* sp. within life cycle stages of the digenean *Plagiorchis elegans*. Appl. Environ. Microbiol. 82, 2356–2362. doi: 10.1128/AEM.04098-15 26873314 PMC4959474

[B59] Gruszczyk J. Kanjee U. Chan L. J. Menant S. Malleret B. Lim N. T. . (2018). Transferrin receptor 1 is a reticulocyte-specific receptor for *Plasmodium vivax*. Science 359, 48–55. doi: 10.1126/science.aan1078 29302006 PMC5788258

[B60] Grybchuk D. Macedo D. H. Kleschenko Y. Kraeva N. Lukashev A. N. Bates P. A. . (2020). The first non-LRV RNA virus in *Leishmania*. Viruses 12, 168. doi: 10.3390/v12020168 32024293 PMC7077295

[B61] Guillén N. (2023). Pathogenicity and virulence of *Entamoeba histolytica*, the agent of amoebiasis. Virulence 14, 2158656. doi: 10.1080/21505594.2022.2158656 36519347 PMC9815260

[B62] Gupta P. Hiller A. Chowdhury J. Lim D. Lim D. Y. Saeij J. P. . (2024). A parasite odyssey: An RNA virus concealed in *Toxoplasma gondii*. Virus Evol. 10, veae040. doi: 10.1093/ve/veae040 38817668 PMC11137675

[B63] Hajialilo E. Rezaeian M. Niyyati M. Pourmand M. R. Mohebali M. Norouzi M. . (2019). Molecular characterization of bacterial, viral and fungal endosymbionts of *Acanthamoeba* isolates in keratitis patients of Iran. Exp. Parasitol. 200, 48–54. doi: 10.1016/j.exppara.2019.03.013 30917916

[B64] Hammoud A. Louni M. Missé D. Cortaredona S. Fenollar F. Mediannikov O. (2022). Phylogenetic relationship between the endosymbiont “Candidatus Riesia pediculicola” and its human louse host. Parasites Vectors 15, 73. doi: 10.1186/s13071-022-05203-z 35248159 PMC8898481

[B65] Hartley M. A. Bourreau E. Rossi M. Castiglioni P. Eren R. O. Prevel F. . (2016). Leishmaniavirus-dependent metastatic leishmaniasis is prevented by blocking IL-17A. PloS Pathog. 12, e1005852. doi: 10.1371/journal.ppat.1005852 27658195 PMC5033371

[B66] Hartley M. A. Ronet C. Zangger H. Beverley S. M. Fasel N. (2012). *Leishmania* RNA virus: when the host pays the toll. Front. Cell. Infect. Microbiol. 12, 99. doi: 10.3389/fcimb.2012.00099 22919688 PMC3417650

[B67] Hassan N. A. Badi F. A. Mohd-Shaharuddin N. Yusoff W. S. Lim Y. A. Chua K. H. . (2022). A conventional multiplex PCR for the detection of four common soil-transmitted nematodes in human feces: development and validation. Trop. Biomed. 39, 135–143. doi: 10.47665/tb.39.1.016 35507936

[B68] He D. Pengtao G. Ju Y. Jianhua L. He L. Guocai Z. . (2017). Differential protein expressions in virus-infected and uninfected *Trichomonas vaginalis*. Korean J. Parasitol. 55, 121. doi: 10.3347/kjp.2017.55.2.121 28506033 PMC5450954

[B69] Hemphill A. Stadelmann B. Rufener R. Spiliotis M. Boubaker G. Müller J. . (2014). Treatment of echinococcosis: albendazole and mebendazole–what else? Parasite 21, 70. doi: 10.1051/parasite/2014073 25526545 PMC4271654

[B70] Herz M. Brehm K. (2019). Evidence for densovirus integrations into tapeworm genomes. Parasites Vectors 12, 560. doi: 10.1186/s13071-019-3820-1 31771643 PMC6880638

[B71] Hruska J. F. Mattern C. F. Diamond L. S. (1974). Viruses of *Entamoeba histolytica* IV. Studies on the nucleic acids of the filamentous and polyhedral viruses. J. Virol. 13, 205–210. doi: 10.1128/jvi.13.1.205-210.1974 4129841 PMC355276

[B72] Hübner M. P. Pfarr K. Hoerauf A. (2022). “ *Wolbachia* endosymbionts as treatment targets for filarial diseases,” in Human and Animal Filariases: Landscape, Challenges, and Control. Eds. Kaminsky R. Geary T. G. (Weinheim: WILEY-VCH), 589–614. doi: 10.1002/9783527823413.ch24

[B73] Janssen M. E. Takagi Y. Parent K. N. Cardone G. Nibert M. L. Baker T. S. (2015). Three-dimensional structure of a protozoal double-stranded RNA virus that infects the enteric pathogen *Giardia lamblia*. J. Virol. 89, 1182–1194. doi: 10.1128/JVI.02745-14 25378500 PMC4300635

[B74] Jenkins T. P. Brindley P. J. Gasser R. B. Cantacessi C. (2019). Helminth microbiomes–a hidden treasure trove? Trends Parasitol. 35, 13–22. doi: 10.1016/j.pt.2018.10.007 30503365

[B75] Jenkins M. C. Higgins J. Abrahante J. E. Kniel K. E. O’Brien C. Trout J. . (2008). Fecundity of *Cryptosporidium parvum* is correlated with intracellular levels of the viral symbiont CPV. Int. J. Parasitol. 38, 1051–1055. doi: 10.1016/j.ijpara.2007.11.005 18096164

[B76] Jenkins M. C. O’brien C. N. Santin M. Fayer R. (2015). Changes in the levels of Cryspovirus during *in vitro* development of *Cryptosporidium parvum*. Parasitol. Res. 114, 2063–2068. doi: 10.1007/s00436-015-4390-6 25704645

[B77] Keeler E. L. Merenstein C. Reddy S. Taylor L. J. Cobián-Güemes A. G. Zankharia U. . (2023). Widespread, human-associated redondoviruses infect the commensal protozoan *Entamoeba gingivalis*. Cell Host Microbe 31, 58–68. doi: 10.1016/j.chom.2022.11.002 36459997 PMC9969835

[B78] Kinsella C. M. Bart A. Deijs M. Broekhuizen P. Kaczorowska J. Jebbink M. F. . (2020). *Entamoeba* and *Giardia* parasites implicated as hosts of CRESS viruses. Nat. Commun. 11, 4620. doi: 10.1038/s41467-020-18474-w 32934242 PMC7493932

[B79] Kirkness E. F. Haas B. J. Sun W. Braig H. R. Perotti M. A. Clark J. M. . (2010). Genome sequences of the human body louse and its primary endosymbiont provide insights into the permanent parasitic lifestyle. Proc. Natl. Acad. Sci. U.S.A. 107, 12168–12173. doi: 10.1073/pnas.1003379107 20566863 PMC2901460

[B80] Kittur N. King C. H. Campbell C. H. Kinung’hi S. Mwinzi P. N. Karanja D. M. . (2019). Persistent hotspots in schistosomiasis consortium for operational research and evaluation studies for gaining and sustaining control of schistosomiasis after four years of mass drug administration of praziquantel. Am. J. Trop. Med. Hyg. 101, 617. doi: 10.4269/ajtmh.19-0193 31287046 PMC6726953

[B81] Klomkliew P. Sawaswong V. Chanchaem P. Nimsamer P. Adisakwattana P. Phuphisut O. . (2022). Gut bacteriome and metabolome of *Ascaris lumbricoides* in patients. Sci. Rep. 12, 19524. doi: 10.1038/s41598-022-23608-9 36376367 PMC9663418

[B82] Kochanowsky J. A. Betts E. L. Encinas G. Amoah J. Johnson P. J. (2025). *Trichomonas vaginalis* extracellular vesicles suppress IFNϵ-mediated responses driven by its intracellular bacterial symbiont *Mycoplasma hominis*. Proc. Natl. Acad. Sci. U.S.A. 122, e2508297122. doi: 10.1073/pnas.2508297122 40560611 PMC12232435

[B83] Kozyk M. Strubchevska K. Fisher J. (2023). *Wohlfahrtiimonas chitiniclastica*: A rare infection reported in an adult with liver cirrhosis. Clin. Case Rep. 11, e6972. doi: 10.1002/ccr3.6972 36846170 PMC9949360

[B84] Kubiak K. Sielawa H. Chen W. Dzika E. (2018). Endosymbiosis and its significance in dermatology. J. Eur. Acad. Dermatol. Venereol. 32, 347–354. doi: 10.1111/jdv.14721 29194776

[B85] Lacey N. Delaney S. Kavanagh K. Powell F. C. (2007). Mite‐related bacterial antigens stimulate inflammatory cells in rosacea. Br. J. Dermatol. 157, 474–481. doi: 10.1111/j.1365-2133.2007.08028.x 17596156

[B86] Lafleur A. Olivier M. (2022). Viral endosymbiotic infection of protozoan parasites: How it influences the development of cutaneous leishmaniasis. PloS Pathog. 18, e1010910. doi: 10.1371/journal.ppat.1010910 36327251 PMC9632828

[B87] Lagunas-Rangel F. A. Kameyama-Kawabe L. Y. Bermúdez-Cruz R. M. (2021). Giardiavirus: an update. Parasitol. Res. 120, 1943–1948. doi: 10.1007/s00436-021-07167-y 33956215

[B88] Lalor R. Cwiklinski K. Calvani N. E. D. Dorey A. Hamon S. Corrales J. L. . (2021). Pathogenicity and virulence of the liver flukes *Fasciola hepatica* and *Fasciola gigantica* that cause the zoonosis Fasciolosis. Virulence 12, 2839–2867. doi: 10.1080/21505594.2021.1996520 34696693 PMC8632118

[B89] Lebbad M. Winiecka-Krusnell J. Stensvold C. R. Beser J. (2021). High diversity of *Cryptosporidium* species and subtypes identified in cryptosporidiosis acquired in Sweden and abroad. Pathogens 10, 523. doi: 10.3390/pathogens10050523 33926039 PMC8147002

[B90] Levecke B. Vlaminck J. Andriamaro L. Ame S. Belizario V. Degarege A. . (2020). Evaluation of the therapeutic efficacy of praziquantel against schistosomes in seven countries with ongoing large-scale deworming programs. Int. J. Parasitol. Drugs Drug Resist. 14, 183–187. doi: 10.1016/j.ijpddr.2020.10.003 33125936 PMC7595844

[B91] Li L. Cao L. Yang Q. Zhao Z. Yuan J. Liu S. . (2025). Giardiavirus infection alleviates growth restriction and intestinal damage caused by the intestinal parasite *Giardia duodenalis*. Parasit Vectors 18, 71. doi: 10.1186/s13071-025-06692-4 39994730 PMC11853539

[B92] Lockard R. D. Wilson M. E. Rodríguez N. E. (2019). Sex‐related differences in immune response and symptomatic manifestations to infection with *Leishmania* species. J. Immunol. Res. 2019, 4103819. doi: 10.1155/2019/4103819 30756088 PMC6348913

[B93] Mahanty S. (2016). Host–parasite interactions and the immunobiology of cestodes. Parasite Immunol. 38, 121–123. doi: 10.1111/pim.12309 26864711

[B94] Maleki-Ravasan N. Ahmadi N. Soroushzadeh Z. Raz A. A. Zakeri S. Dinparast Djadid N. (2020). New insights into culturable and unculturable bacteria across the life history of medicinal maggots *Lucilia sericata* (Meigen)(Diptera: Calliphoridae). Front. Microbiol. 11, 505. doi: 10.3389/fmicb.2020.00505 32322242 PMC7156559

[B95] Manny A. R. Hetzel C. A. Mizani A. Nibert M. L. (2022). Discovery of a novel species of trichomonasvirus in the human parasite *Trichomonas vaginalis* using transcriptome mining. Viruses 14, 548. doi: 10.3390/v14030548 35336955 PMC8953718

[B96] Manoj R. R. Latrofa M. S. Epis S. Otranto D. (2021). *Wolbachia*: endosymbiont of onchocercid nematodes and their vectors. Parasites Vectors 14, 245. doi: 10.1186/s13071-021-04742-1 33962669 PMC8105934

[B97] Margarita V. Rappelli P. Dessi D. Pintus G. Hirt R. P. Fiori P. L. (2016). Symbiotic association with *Mycoplasma hominis* can influence growth rate, ATP production, cytolysis and inflammatory response of *Trichomonas vaginalis*. Front. Microbiol. 7, 953. doi: 10.3389/fmicb.2016.00953 27379081 PMC4913105

[B98] Martin A. M. Fraser T. A. Lesku J. A. Simpson K. Roberts G. L. Garvey J. . (2018). The cascading pathogenic consequences of *Sarcoptes scabiei* infection that manifest in host disease. R. Soc Open Sci. 5, 180018. doi: 10.1098/rsos.180018 29765692 PMC5936957

[B99] Martin B. D. Schwab E. (2013). Current usage of symbiosis and associated terminology. Int. J. Biol. 5, 32–45. doi: 10.5539/ijb.v5n1p32

[B100] Marucci G. Zullino I. Bertuccini L. Camerini S. Cecchetti S. Pietrantoni A. . (2021). Re-discovery of giardiavirus: genomic and functional analysis of viruses from *Giardia duodenalis* isolates. Biomedicines 9, 654. doi: 10.3390/biomedicines9060654 34201207 PMC8230311

[B101] Marzook N. B. Sateriale A. (2020). Crypto-currency: investing in new models to advance the study of *Cryptosporidium* infection and immunity. Front. Cell. Infect. Microbiol. 10, 587296. doi: 10.3389/fcimb.2020.587296 33312965 PMC7708325

[B102] Mattern C. F. Keister D. B. Daniel W. A. Diamond L. S. Kontonis A. D. (1977). Viruses of *Entamoeba histolytica*. VII. Novel beaded virus. J. Virol. 23, 685–691. doi: 10.1128/JVI.23.3.685-691.1977 197265 PMC515880

[B103] Mawa P. A. Kincaid-Smith J. Tukahebwa E. M. Webster J. P. Wilson S. (2021). Schistosomiasis morbidity hotspots: roles of the human host, the parasite and their interface in the development of severe morbidity. Front. Immunol. 12, 635869. doi: 10.3389/fimmu.2021.635869 33790908 PMC8005546

[B104] Mazhari R. Takashima E. Longley R. J. Ruybal-Pesantez S. White M. T. Kanoi B. N. . (2023). Identification of novel *Plasmodium vivax* proteins associated with protection against clinical malaria. Front. Cell. Infect. Microbiol. 13, 1076150. doi: 10.3389/fcimb.2023.1076150 36761894 PMC9905245

[B105] McNulty S. N. Tort J. F. Rinaldi G. Fischer K. Rosa B. A. Smircich P. . (2017). Genomes of *Fasciola hepatica* from the Americas reveal colonization with *Neorickettsia* endobacteria related to the agents of Potomac horse and human Sennetsu fevers. PloS Genet. 13, e1006537. doi: 10.1371/journal.pgen.1006537 28060841 PMC5257007

[B106] Mercer F. Johnson P. J. (2018). *Trichomonas vaginalis*: pathogenesis, symbiont interactions, and host cell immune responses. Trends Parasitol. 34, 683–693. doi: 10.1016/j.pt.2018.05.006 30056833 PMC11132421

[B107] Mirabedini Z. Mirjalali H. Kazemirad E. Khamesipour A. Samimirad K. Koosha M. . (2023). The effects of *Leishmania* RNA virus 2 (LRV2) on the virulence factors of *L. major* and pro-inflammatory biomarkers: an *in vitro* study on human monocyte cell line (THP-1). BMC Microbiol. 23, 398. doi: 10.1186/s12866-023-03140-0 38097942 PMC10720061

[B108] Miska K. B. Jenkins M. C. Trout J. M. Santín M. Fayer R. (2009). Detection and comparison of Giardia virus (GLV) from different assemblages of *Giardia duodenalis*. J. Parasitol. 95, 1197–1200. doi: 10.1645/GE-1876.1 19320541

[B109] Mkandawire T. T. Sateriale A. (2022). The long and short of next generation sequencing for *Cryptosporidium* research. Front. Cell. Infect. Microbiol. 12, 871860. doi: 10.3389/fcimb.2022.871860 35419299 PMC8995782

[B110] Molyneux D. H. (1974). Virus-like particles in *Leishmania* parasites. Nature 249, 588–589. doi: 10.1038/249588a0 4834085

[B111] Mungroo M. R. Siddiqui R. Khan N. A. (2021). War of the microbial world: *Acanthamoeba* spp. interactions with microorganisms. Folia Microbiol. 66, 689–699. doi: 10.1007/s12223-021-00889-7 34145552 PMC8212903

[B112] Muslim A. Aazmi S. Er Y. X. Shahrizal S. Lim Y. A. L. (2024). *Ascaris lumbricoides* harbors a distinct gut microbiota profile from its human host: preliminary insights. Food Waterborne Parasitol. 34, e00223. doi: 10.1016/j.fawpar.2024.e00223 38323096 PMC10844936

[B113] Parmentier L. Cusini A. Müller N. Zangger H. Hartley M. A. Desponds C. . (2016). Severe cutaneous leishmaniasis in a human immunodeficiency virus patient coinfected with *Leishmania braziliensis* and its endosymbiotic virus. Am. J. Trop. Med. Hyg. 94, 840. doi: 10.4269/ajtmh.15-0803 26834198 PMC4824227

[B114] Petrone M. E. Charon J. Parry R. H. Grigg M. J. Piera K. A. Westaway J. A. . (2026). Mapping the global distribution and spread of the Plasmodium vivax-associated virus MaRNAV-1. Virus Evol. 12, veag031. doi: 10.1093/ve/veag031 42311450 PMC13271372

[B115] Phan T. G. Mori D. Deng X. Rajindrajith S. Ranawaka U. Ng T. F. . (2015). Small circular single stranded DNA viral genomes in unexplained cases of human encephalitis, diarrhea, and in untreated sewage. Virology 482, 98–104. doi: 10.1016/j.virol.2015.03.011 25839169 PMC4461510

[B116] Price C. T. Hanford H. E. Al-Quadan T. Santic M. Shin C. J. Da'as M. S. . (2024). Amoebae as training grounds for microbial pathogens. MBio 15, e00827-24. doi: 10.1128/mbio.00827-24 38975782 PMC11323580

[B117] Queiroz V. F. Rodrigues R. A. de Miranda Boratto P. V. La Scola B. Andreani J. Abrahão J. S. (2022). Amoebae: hiding in plain sight: unappreciated hosts for the very large viruses. Annu. Rev. Virol. 9, 79–98. doi: 10.1146/annurev-virology-100520-125832 35655338

[B118] Quek S. Cook D. A. Wu Y. Marriott A. E. Steven A. Johnston K. L. . (2022). *Wolbachia* depletion blocks transmission of lymphatic filariasis by preventing chitinase-dependent parasite exsheathment. Proc. Natl. Acad. Sci. U.S.A. 119, e2120003119. doi: 10.1073/pnas.2120003119 35377795 PMC9169722

[B119] Rada P. Hrdý I. Zdrha A. Narayanasamy R. K. Smutná T. Horáčková J. . (2022). Double-stranded RNA viruses are released from *Trichomonas vaginalis* inside small extracellular vesicles and modulate the exosomal cargo. Front. Microbiol. 13, 893692. doi: 10.3389/fmicb.2022.893692 35602021 PMC9114709

[B120] Raina J. B. Eme L. Pollock F. J. Spang A. Archibald J. M. Williams T. A. (2018). Symbiosis in the microbial world: from ecology to genome evolution. Biol. Open 7, bio032524. doi: 10.1242/bio.032524 29472284 PMC5861367

[B121] Ramaiah K. D. Ottesen E. A. (2014). Progress and impact of 13 years of the global programme to eliminate lymphatic filariasis on reducing the burden of filarial disease. PloS Negl.Trop. Dis. 8, e3319. doi: 10.1371/journal.pntd.0003319 25412180 PMC4239120

[B122] Rayamajhee B. Sharma S. Willcox M. Henriquez F. L. Rajagopal R. N. Shrestha G. S. . (2022). Assessment of genotypes, endosymbionts and clinical characteristics of *Acanthamoeba* recovered from ocular infection. BMC Infect. Dis. 22, 757. doi: 10.1186/s12879-022-07741-4 36175838 PMC9520893

[B123] Reed K. Reynolds S. B. Smith C. (2021). The first case of Ignatzschineria ureiclastica/larvae in the United States presenting as a myiatic wound infection. Cureus 13, e14518. doi: 10.7759/cureus.14518 34007769 PMC8126268

[B124] Ríos-Valencia D. G. Ambrosio J. Tirado-Mendoza R. Carrero J. C. Laclette J. P. (2023). What about the cytoskeletal and related proteins of tapeworms in the host’s immune response? An integrative overview. Pathogens 12, 840. doi: 10.3390/pathogens12060840 37375530 PMC10302083

[B125] Risch F. Kazakov A. Specht S. Pfarr K. Fischer P. U. Hoerauf A. . (2024). The long and winding road towards new treatments against lymphatic filariasis and onchocerciasis. Trends Parasitol. 40, 829–845. doi: 10.1016/j.pt.2024.07.005 39122645

[B126] Rojas-Lopez L. Elwin K. Chalmers R. M. Enemark H. L. Beser J. Troell K. (2020). Development of a gp60-subtyping method for *Cryptosporidium felis*. Parasites Vectors 13, 39. doi: 10.1186/s13071-020-3906-9 31973771 PMC6979280

[B127] Rossi M. Fasel N. (2018). How to master the host immune system? *Leishmania* parasites have the solutions! Int. Immunol. 30, 103–111. doi: 10.1093/intimm/dxx075 29294040 PMC5892169

[B128] Saberi R. Fakhar M. Mohebali M. Anvari D. Gholami S. (2019). Global status of synchronizing *Leishmania* RNA virus in *Leishmania* parasites: A systematic review with meta‐analysis. Transbound Emerg. Dis. 66, 2244–2251. doi: 10.1111/tbed.13316 31376334

[B129] Sabeti S. Koosha M. Kazemirad E. Mirabedini Z. Mohebali M. Saberi R. . (2025). Transcriptional alterations of virulence factors in *Leishmania major* clinical isolates harboring *Leishmania* RNA virus 2 (LRV2). BMC Infect. Dis. 25, 319. doi: 10.1186/s12879-025-10717-9 40050765 PMC11884114

[B130] Sakhaee F. Mosayebi Amroabadi J. Razi S. Vaziri F. Abdolrahimi F. Moghaddam S. . (2022). Detection of Mimivirus from respiratory samples in tuberculosis-suspected patients. Sci. Rep. 12, 8676. doi: 10.1038/s41598-022-12757-6 35606506 PMC9126102

[B131] Schäfer C. Zanghi G. Vaughan A. M. Kappe S. H. (2021). *Plasmodium vivax* latent liver stage infection and relapse: biological insights and new experimental tools. Annu. Rev. Microbiol. 75, 87–106. doi: 10.1146/annurev-micro-032421-061155 34196569

[B132] Schröttner P. Rudolph W. W. Damme U. Lotz C. Jacobs E. Gunzer F. (2017). *Wohlfahrtiimonas chitiniclastica*: current insights into an emerging human pathogen. Epidemiol. Infect. 145, 1292–1303. doi: 10.1017/S0950268816003411 28162132 PMC9203347

[B133] Setegn A. Amare G. A. Mihret Y. (2024). *Wolbachia* and lymphatic filarial nematodes and their implications in the pathogenesis of the disease. J. Parasitol. Res. 2024, 3476951. doi: 10.1155/2024/3476951 38725798 PMC11081757

[B134] Shaw D. Gentekaki E. Tsaousis A. D. (2026). The microbiome within a microbe: Rethinking *Blastocystis* biology. J. Eukaryot. Microbiol. 73, e70056. doi: 10.1111/jeu.70056 41588828 PMC12835684

[B135] Siddiqui R. Khan N. A. (2012). Biology and pathogenesis of *acanthamoeba*. Parasites Vectors 5, 6. doi: 10.1186/1756-3305-5-6 22229971 PMC3284432

[B136] Singh N. Chikara S. Sundar S. (2013). SOLiDTM sequencing of genomes of clinical isolates of *Leishmania donovani* from India confirm *Leptomonas* co-infection and raise some key questions. PloS One 8, e55738. doi: 10.1371/journal.pone.0055738 23418454 PMC3572117

[B137] Skinner D. E. Rinaldi G. Koziol U. Brehm K. Brindley P. J. (2014). How might flukes and tapeworms maintain genome integrity without a canonical piRNA pathway? Trends Parasitol. 30, 123–129. doi: 10.1016/j.pt.2014.01.001 24485046 PMC3941195

[B138] Slatko B. E. Taylor M. J. Foster J. M. (2010). The *Wolbachia* endosymbiont as an anti-filarial nematode target. Symbiosis 51, 55–65. doi: 10.1007/s13199-010-0067-1 20730111 PMC2918796

[B139] Stepek G. Buttle D. J. Duce I. R. Behnke J. M. (2006). Human gastrointestinal nematode infections: are new control methods required? Int. J. Exp. Pathol. 87, 325–341. doi: 10.1111/j.1365-2613.2006.00495.x 16965561 PMC2517378

[B140] Sukla S. Nath H. Kamran M. Ejazi S. A. Ali N. Das P. . (2022). Detection of *Leptomonas seymouri* narna-like virus in serum samples of visceral leishmaniasis patients and its possible role in disease pathogenesis. Sci. Rep. 12, 14436. doi: 10.1038/s41598-022-18526-9 36002553 PMC9402534

[B141] Sunantaraporn S. Sanprasert V. Pengsakul T. Phumee A. Boonserm R. Tawatsin A. . (2015). Molecular survey of the head louse *Pediculus humanus* capitis in Thailand and its potential role for transmitting *Acinetobacter* spp. Parasites Vectors 8, 127. doi: 10.1186/s13071-015-0742-4 25889008 PMC4347573

[B142] Swe P. M. Zakrzewski M. Waddell R. Sriprakash K. S. Fischer K. (2019). High-throughput metagenome analysis of the *Sarcoptes scabiei* internal microbiota and in-situ identification of intestinal *Streptomyces* sp. Sci. Rep. 9, 11744. doi: 10.1038/s41598-019-47892-0 31409870 PMC6692375

[B143] Tatu A. L. Ionescu M. A. Cristea V. C. (2017). *Demodex folliculorum* associated *Bacillus pumilus* in lesional areas in rosacea. Indian J. Dermatol. Venereol. Leprol. 83, 610. doi: 10.4103/ijdvl.IJDVL_921_16 28749385

[B144] Taylor M. J. Bandi C. Hoerauf A. (2005). *Wolbachia*. Bacterial endosymbionts of filarial nematodes. Adv. Parasitol. 60, 245–284. doi: 10.1016/S0065-308X(05)60004-8 16230105

[B145] Taylor M. Mediannikov O. Raoult D. Greub G. (2012). Endosymbiotic bacteria associated with nematodes, ticks and amoebae. FEMS Immunol. Med. Microbiol. 64, 21–31. doi: 10.1111/j.1574-695X.2011.00916.x 22126456

[B146] Thakur L. Kushwaha H. R. Negi A. Jain A. Jain M. (2020). *Leptomonas seymouri* co-infection in cutaneous leishmaniasis cases caused by *Leishmania donovani* from Himachal Pradesh, India. Front. Cell. Infect. Microbiol. 10, 345. doi: 10.3389/fcimb.2020.00345 32760679 PMC7373763

[B147] Thornley S. Marshall R. Jarrett P. Sundborn G. Reynolds E. Schofield G. (2018). Scabies is strongly associated with acute rheumatic fever in a cohort study of Auckland children. J. Paediatr. Child Health 54, 625–632. doi: 10.1111/jpc.13851 29442387

[B148] Toth E. M. Schumann P. Borsodi A. K. Keki Z. Kovacs A. L. Marialigeti K. (2008). *Wohlfahrtiimonas chitiniclastica* gen. nov. sp. nov. a new gammaproteobacterium isolated from *Wohlfahrtia magnifica* (Diptera: Sarcophagidae). Int. J. Syst. Evol. Microbiol. 58, 976–981. doi: 10.1099/ijs.0.65324-0 18398205

[B149] Tsai I. J. Zarowiecki M. Holroyd N. Garciarrubio A. Sanchez-Flores A. Brooks K. L. . (2013). The genomes of four tapeworm species reveal adaptations to parasitism. Nature 496, 57–63. doi: 10.1038/nature12031 23485966 PMC3964345

[B150] Turner J. D. Langley R. S. Johnston K. L. Egerton G. Wanji S. Taylor M. J. (2006). *Wolbachia* endosymbiotic bacteria of *Brugia malayi* mediate macrophage tolerance to TLR-and CD40-specific stimuli in a MyD88/TLR2-dependent manner. J. Immunol. 177, 1240–1249. doi: 10.4049/jimmunol.177.2.1240 16818783

[B151] Ung L. Stothard J. R. Phalkey R. Azman A. S. Chodosh J. Hanage W. P. . (2021). Towards global control of parasitic diseases in the Covid-19 era: One Health and the future of multisectoral global health governance. Adv. Parasitol. 114, 1–26. doi: 10.1016/bs.apar.2021.08.007 34696842 PMC8503781

[B152] Wakid M. H. Almakki A. A. Alsahaf N. S. Almatrafi M. A. (2022). Furuncular myiasis by *Wohlfahrtia magnifica* (Diptera: Sarcophagidae) in a healthy child. Asian Pac. J. Trop. Med. 15, 87–89. doi: 10.4103/1995-7645.338433

[B153] Wang T. Gasser R. B. Korhonen P. K. Young N. D. Ang C. S. Williamson N. A. . (2022). Proteomic analysis of *Sarcoptes scabiei* reveals that proteins differentially expressed between eggs and female adult stages are involved predominantly in genetic information processing, metabolism and/or host-parasite interactions. PloS Negl.Trop. Dis. 16, e0010946. doi: 10.1371/journal.pntd.0010946 36472966 PMC9725168

[B154] Wangwiwatsin A. Kulwong S. Phetcharaburanin J. Namwat N. Klanrit P. Loilome W. . (2023). Toward novel treatment against filariasis: Insight into genome-wide co-evolutionary analysis of filarial nematodes and *Wolbachia*. Front. Microbiol. 14, 1052352. doi: 10.3389/fmicb.2023.1052352 37032902 PMC10073474

[B155] Wei F. Li L. Kong Y. Yan X. Varghese K. Zhang S. . (2024). Evidence for the clinical association between *Demodex* and rosacea: a review. Dermatology 240, 95–102. doi: 10.1159/000534245 37848012

[B156] Wiles T. J. Guillemin K. (2019). The other side of the coin: what beneficial microbes can teach us about pathogenic potential. J. Mol. Biol. 431, 2946–2956. doi: 10.1016/j.jmb.2019.05.001 31078557

[B157] Zangger H. Hailu A. Desponds C. Lye L. F. Akopyants N. S. Dobson D. E. . (2014). *Leishmania aethiopica* field isolates bearing an endosymbiontic dsRNA virus induce pro-inflammatory cytokine response. PloS Negl.Trop. Dis. 8, e2836. doi: 10.1371/journal.pntd.0002836 24762979 PMC3998932

[B158] Zhang Z. Deng Y. Sheng W. Song X. Li Y. Li F. . (2023). The interaction between adhesion protein 33 (TvAP33) and BNIP3 mediates the adhesion and pathogenicity of *Trichomonas vaginalis* to host cells. Parasites Vectors 16, 210. doi: 10.1186/s13071-023-05798-x 37344876 PMC10286359

[B159] Zhang X. Kim C. Y. Worthen T. Witola W. H. (2018). Morpholino-mediated *in vivo* silencing of *Cryptosporidium parvum* lactate dehydrogenase decreases oocyst shedding and infectivity. Int. J. Parasitol. 48, 649–656. doi: 10.1016/j.ijpara.2018.01.005 29530646 PMC6018611

[B160] Zhang Y. Li J. Lai X. Wang Y. Li X. Ren G. . (2025). Molecular characterization and zoonotic potential of *Blastocystis* subtypes in domestic pigs and cattle from Hainan, a tropical island province in China. Parasite 32, 77. doi: 10.1051/parasite/2025070 41348956 PMC12680373

[B161] Zhang H. Zhao C. Zhang X. Li J. Gong P. Wang X. . (2023). A potential role for *Giardia* chaperone protein GdDnaJ in regulating *Giardia* proliferation and Giardiavirus replication. Parasites Vectors 16, 168. doi: 10.1186/s13071-023-05787-0 37226181 PMC10210397

[B162] Zhao L. Rosario K. Breitbart M. Duffy S. (2019). Eukaryotic circular rep-encoding single-stranded DNA (CRESS DNA) viruses: ubiquitous viruses with small genomes and a diverse host range. Adv. Virus Res. 103, 71–133. doi: 10.1016/bs.aivir.2018.10.001 30635078

[B163] Zug R. Hammerstein P. (2012). Still a host of hosts for *Wolbachia*: analysis of recent data suggests that 40% of terrestrial arthropod species are infected. PloS One 7, e38544. doi: 10.1371/journal.pone.0038544 22685581 PMC3369835

